# Evolution and Expression Characteristics of Receptor-Like Cytoplasmic Protein Kinases in *Maize*, *Rice* and *Arabidopsis*

**DOI:** 10.3390/ijms19113680

**Published:** 2018-11-21

**Authors:** Mingxia Fan, Wenjuan Ma, Chen Liu, Chunyu Zhang, Suwen Wu, Meiming Chen, Kuichen Liu, Fengchun Cai, Feng Lin

**Affiliations:** 1 Biotechnology and Bioscience College, Shenyang Agricultural University, 120 Dongling Road, Shenyang 110866, China; 20152166@stu.syau.edu.cn (W.M.); liuchen@syau.edu.cn (C.L.); 1994500024@syau.edu.cn (C.Z.); 20162164@stu.syau.edu.cn (M.C.); 2017220164@stu.syau.edu.cn (K.L.); 2017220160@stu.syau.edu.cn (F.C.); 2 Liaoning Key Laboratory of Urban Integrated Pest Management and Ecological Security, College of Life Science and Engineering Shenyang University, Shenyang 110044, China; syndlc@outlook.com; 3 College of Science Institute, Shenyang Agricultural University No. 120 Dongling Road, Shenyang 110866, China; 2001500072@syau.edu.cn

**Keywords:** *RLCK* gene, duplication, synteny, kinase activity site, negative selection

## Abstract

Receptor-like cytoplasmic protein kinases (RLCKs) are involved in various activities in plant growth and development. We have totally identified 162, 160, and 402 *RLCK* genes in maize, rice, and Arabidopsis genomes, respectively. Phylogenetic analyses divided 724 *RLCK* genes into 15 subfamilies and similar structural patterns of kinase activity sites and functional sites were observed within the subfamilies. Furthermore, the structural patterns of intron/exon in the same subfamilies were similar, implicating their close evolutionary relationship. Chromosome distribution indicated that segmental duplication of *RLCK* genes might be a major mechanism contributing to the expansion of the RLCK superfamilies in maize, rice, and Arabidopsis, respectively. The analysis of the synteny relationship and gene structure indicated that the evolution of most RLCKs in maize were prior to rice and Arabidopsis. Most of the ratio of *Ka*/*Ks* is inferior to one, suggesting that *RLCK* genes have experienced the negative selection in maize, rice and Arabidopsis. Duplication time revealed that the maize was the earliest emergence among these three species. The expression profiles showed that there are some specifically expressed *RLCK* genes in maize root, leaf, ear, and tassel. These specific expression genes may participate in the developmental regulation of these maize tissues. Our results will be useful in providing new insights into evolution of RLCKs and revealing the regulatory network of maize, rice, and Arabidopsis development.

## 1. Introduction

Receptor-like protein kinases (RLKs), widely existing in plants, are kinds of enzymes which recognize the signals by extracellular domains, and transfer the signals to intracellular domains through the transmembrane domains [1]. According to domains, RLKs can be classified into receptor-like protein kinases (RLP), transmembrane receptor kinases (TMRK), and receptor-like cytoplasmic protein kinases (RLCKs). RLCKs lack transmembrane domains and extracellular domains, only containing intracellular domains. Previous studies revealed that there were approximately 25% and 17% of RLKs members belonging to RLCKs in Arabidopsis and rice, respectively [2,3,4]. RLKs can regulate plant development, growth and fertilization, self-incompatibility [5,6].

The first RLK gene in maize was cloned by Walker and Zhang [7]. After that, a large number of RLKs, including RLCKs, have been found successively in plants. Yang et al. [8] reported that A novel *CRCK1* in Arabidopsis was up-regulated expression under the abiotic stress treatment, suggested that *CRCK1* may be involved in various stress-resistance signal transduction pathways. The novel RLCKs NtPK1 and NtPK2 could regulate the signaling pathways of pollen germination and pollen tube growth [9]. The triggered stomata immunity by pathogen-associated molecular patterns can be mediated through BIK1 [10]. OsRLCK102 regulates development by Xa21-mediated immunity in BR signaling pathway [11]. GsRLCK acted as an important function in response to abscisic acid (ABA), drought and salt stresses [12]. Hence, RLCKs may play important roles in stress-resistance, diseases-resistance, and developmental regulation in plants.

Here intron numbers, the distribution of genes on chromosomes, and the evolutionary characteristics of *RLCK* genes, combining with the genes synteny relations among maize, rice, and Arabidopsis, were illustrated. The RLCK superfamilies are expanded due to segmental duplications among maize, Arabidopsis, and rice chromosomes. Meanwhile, the ratio of *Ka/Ks* and the duplication time among these three species showed that *RLCK* genes in monocotyledons have undergone a longer evolutionary time than dicotyledons. Meanwhile, *RLCK* genes expression profiles and sequence analysis highlighted their potential functional diversity.

## 2. Results

### 2.1. Identification of RLCKs in Maize, Rice and Arabidopsis

The RLKs, including the RLCK subfamily, were searched in the databases of maize, rice, and Arabidopsis databases and the relevant articles. A total of 2449 sequences were retrieved and regarded as “potential” RLKs. The sub-cellular localization of all obtained RLKs was predicted. After removing the incomplete sequences manually, we totally retrieved 724 “candidate” RLCKs, including 162, 160, and 402 in maize, rice, and Arabidopsis, respectively (Tables S1 and S2). 

### 2.2. Conservative Evolution of RLCKs in Maize, Rice and Arabidopsis

**Figure 1:**
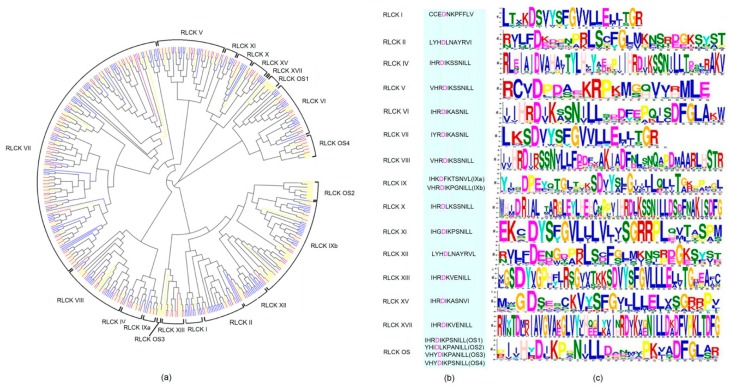
The phylogram and conserved kinase domain analysis of receptor-like cytoplasmic protein kinases (RLCKs) in maize, rice, and Arabidopsis. (**a**) The RLCK phylogram of maize, rice, and Arabidopsis. The pinkish purple, yellow, and blue were used to show the *RLCK* genes from maize, rice, and Arabidopsis, respectively. (**b**) The conserved kinase domain of RLCKs in each group. The pink letters were the predicted kinase activity sites of RLCKs. (**c**) The RLCK conservative motifs in each group.

A total of 724 RLCK kinase domain sequences in maize, rice, and Arabidopsis were aligned and 15 groups were generated (Figure 1a). Each group was named after the *RLCK* gene classification according to the “PlantsP Kinase Classification” database (http://plantsp.genomics.purdue.edu/). Most *RLCK* genes were classified into RLCK V~RLCK IX groups. RLCK OS only contained maize and rice *RLCK* genes, indicating the differentiation between monocotyledons and dicotyledons during the evolution. Based on conservative motifs of kinase domains, the genetic distance of some genes in RLCK VII was more closely with RLCK OS1 and RLCK OS4, indicating that kinase domains among RLCK VII, RLCK OS1, and RLCK OS4 were similar and conservative, but other domains may be different (Figure 1a). It was suggested that *RLCK* genes with a conservative structure may perform a similar function in plants. It is also implied that *RLCK* genes had a common ancestor during the evolution. The predicted kinase activity sites were shown in Figure 1b. The kinase domains between the adjacent groups were conservative and the kinase activity sites were contained in the conserved sequences. We found that most RLCK kinase activity sites were aspartic acid (d) (Figure 1b). The aspartic acid (d) was also discovered between the fourth and fifth amino acid in all the RLCK motifs (Figure 1c). These results showed the RLCK functional sites may be the same amino acid between the different groups. In Figure 1c, the different groups were shown to be significantly conservative, such as RLCK II and RLCK XII, RLCK VII, and RLCK OS. RLCK VII and RLCK OS motifs were well conserved over the first 15 and the last six amino acid residues. Above all, the RLCK amino acid sequences were well conserved in the period of evolution in maize, rice, and Arabidopsis.

**Figure 2:**
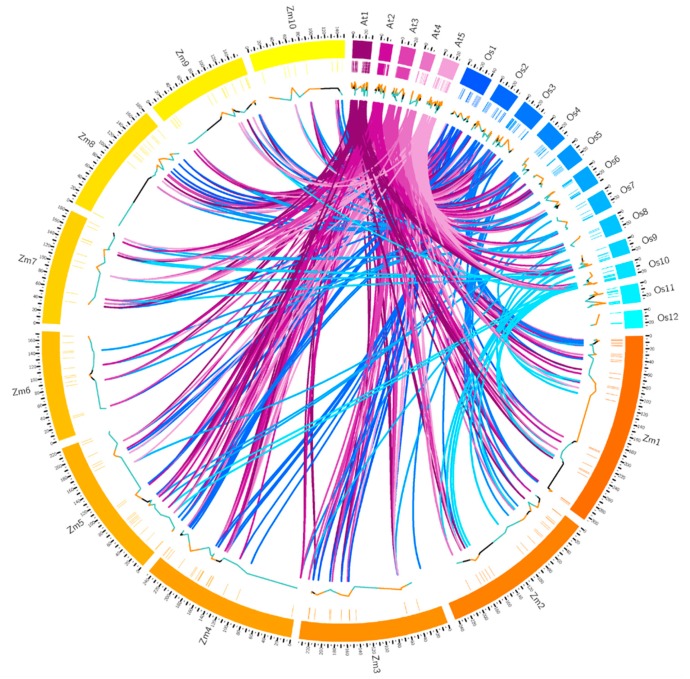
Circos map of *RLCK* genes in maize, rice and Arabidopsis. The outer two circles were proportional chromosomes for maize, rice, and Arabidopsis in megabase (Mb) units. The third circle represented each *RLCK* gene distribution on chromosomes. Lines below each chromosome represent the intron numbers of *RLCK* genes (orange > 5 introns, black = 5 introns, blue < 5 introns). Innermost colored lines represent synteny gene pairs among the genomes of maize, rice, and Arabidopsis.

The conservation of gene structure, related with the number of introns, plays a crucial role in the occurrence of evolution [13]. Thus, we analyzed the intron numbers and the distribution of *RLCK* genes of maize, rice and Arabidopsis. In Figure 2, the third circle is the statistics of intron numbers ranging from 0 to 15. There were 50, 67, and 123 *RLCK* genes with more than five introns; there were 25, 19, and 46 *RLCK* genes with five introns; 86, 49, and 233 *RLCK* genes with less than five introns in maize, rice, and Arabidopsis, respectively.

### 2.3. The Evolution Selection of RLCKs in Maize, Rice and Arabidopsis

**Figure 3:**
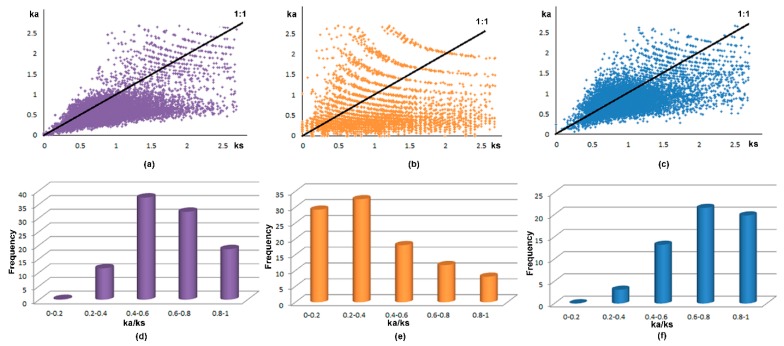
The distribution and frequency of *Ka/Ks* values. The purple, blue, and orange colors represent the maize, rice, and Arabidopsis, respectively. (**a**), (**b**) and (**c**) show the *Ka/Ks* distribution. The line in black is the boundary of *Ka/Ks* = 1. (**d**), (**e**), and (**f**) represent the frequency distribution of *Ka/Ks* < 1.

To understand the evolutionary characteristics, the synonymous mutations (*Ks*) and non-synonymous mutations (*Ka*) were calculated. It is generally believed that the value of Ks was not affected by natural selection, but the *Ka* was affected by natural selection. The *Ka/Ks* value can also explain positive selection (*Ka/Ks* > 1), neutral selection (*Ka/Ks* = 1) and negative selection (*Ka/Ks* < 1) during the evolution [14,15]. The ratio distribution of each pair was shown in Figure 3a–c. The percent of *Ka/Ks* < 1 were 86%, 67% and 70% in these three species, respectively. The negative selection of maize *RLCK* genes (*Ka/Ks* < 1) was higher than Arabidopsis and rice. These results illustrated that *RLCK* genes in these three species were undergoing the negative selection and the monocotyledons maybe performed more negative selection than dicotyledons during the evolution. The maize *Ka/Ks* frequency mainly distributed between 0.4 and 0.8, rice between 0 and 0.4, and Arabidopsis between 0.6 and 1 (Figure 3d–f). Thess results declared that the negative selection in maize was stronger than Arabidopsis but weaker than rice. The duplication time (T) of *RLCK* genes is from 7.1 to 153.7 Mya ago in maize, from 5.5 to 66.5 Mya in Arabidopsis, and from 0.15 to 151.3 Mya in rice, suggesting that the maize *RLCK* genes have been undergone a longer evolutionary time than Arabidopsis and rice.

### 2.4. Segmental Duplication of RLCKs in Maize, Rice and Arabidopsis

**Figure 4:**
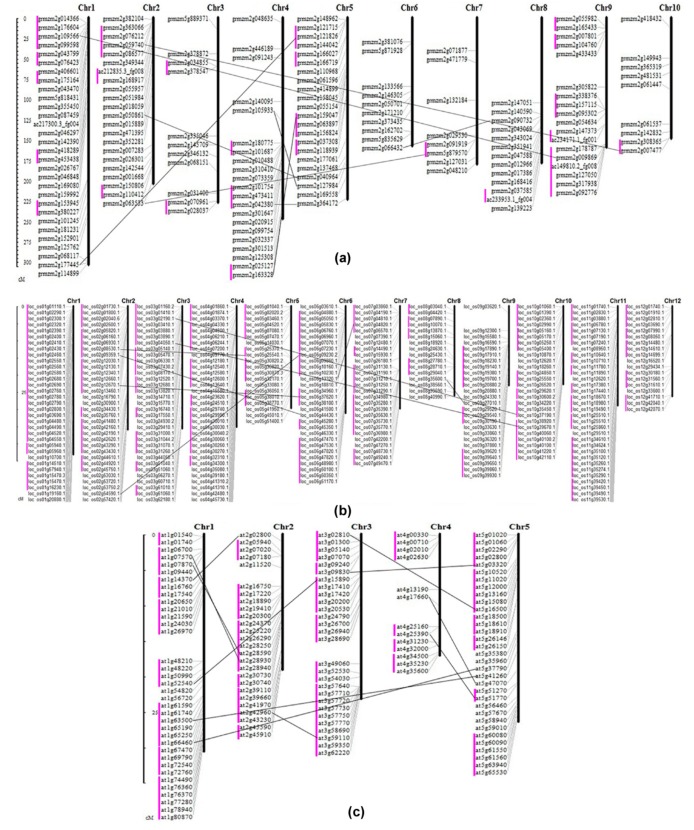
*RLCK* gene distribution on chromosomes in maize (**a**), rice (**b**), and Arabidopsis (**c**). The lines in purple represent gene clusters.

The gene duplication events were significantly occurred in the plants more than other eukaryotes [13]. The gene duplication mainly includes whole genome duplication (WED), segmental duplication, tandem duplication, and transposon-mediated duplication [16,17]. There were nine, eight, and eleven segmental duplication events founded in maize, rice, and Arabidopsis, respectively (Figure 4). These results suggested that the segmental duplication played an important role during the *RLCK* genes evolution. Gene cluster refers to the genes within a 2 MB region in a chromosome [18]. In Figure 4, there were 25 gene clusters in maize, less than 30 and 63 in Arabidopsis and rice, respectively.

### 2.5. The Distribution and Synteny of RLCK Genes in Maize, Rice and Arabidopsis

To understand the chromosomal distributions of *RLCK* genes, MapInspect software was employed to analyze the *RLCK* gene starting positions on chromosomes (Figure 4). Maize *RLCK* genes were unevenly distributed on 10 chromosomes (Figure 4a). On chromosome 3, 7, and 10, there were the minimum number of genes (10 genes) and the maximum number was on chromosome 1 which more evenly distributed 29 genes. The 162 maize *RLCK* genes have showed a high density in the terminal of chromosome 1, 2, and 9 and on the end of chromosome 4, 6, 8, and 9. In Figure 4b,c, the rice and Arabidopsis *RLCK* genes were more evenly distributed than maize. 

The innermost map showed the synteny relations of *RLCK* genes among maize, rice, and Arabidopsis (Figure 2). In total, 106 *RLCK* genes in maize showed synteny relationships with Arabidopsis and rice, indicating that most *RLCK* genes in maize were conservative during the evolution comparing with Arabidopsis and rice, and, further, these three species shared common ancestors.

### 2.6. Dynamic Expression of RLCK Genes in Maize

**Figure 5:**
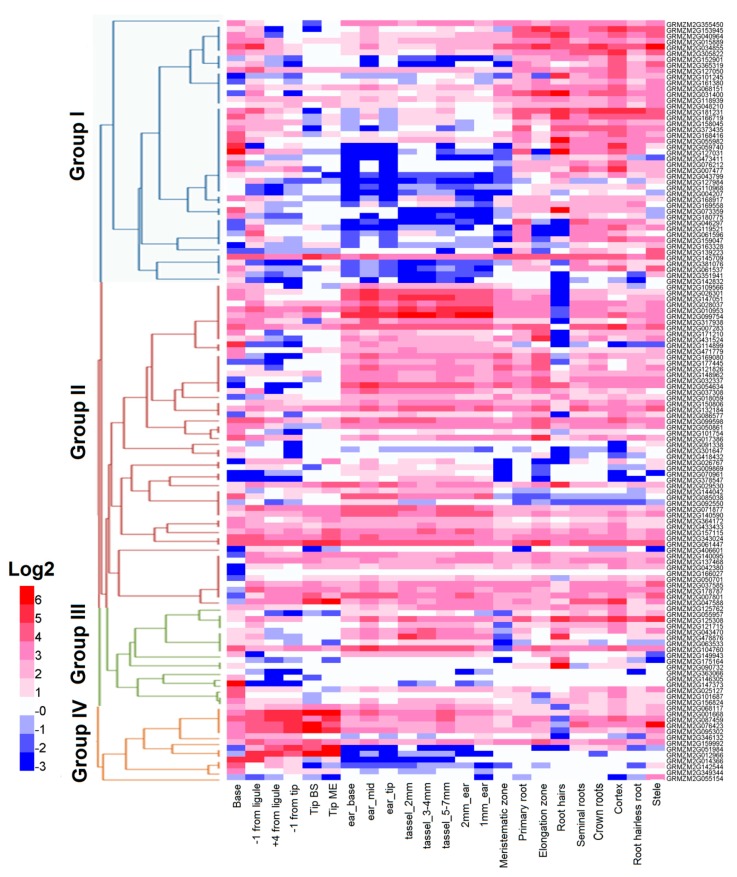
Maize *RLCK* gene hierarchial clustering. On the left, the color scale indicates the log2 values and the hierarchial clustering displays four expression patterns marked by different colors. The different development periods of leaf, root, ear, and tassel in maize are showed on the bottom.

Figure 5 showed the maize *RLCK* genes expression profile based on 131 maize *RLCK* genes. Table S3 listed *RLCK* genes expression data from the Maize eFP Browser [19]. The other 31 maize *RLCK* genes, lacking expression data, were not shown in the expression profile. Leaf, root, ear, and tassel in different development stages displayed in the bottom of the hierarchial clustering heatmap. Based on expression data, maize *RLCK* genes were classified into four groups. There were 45 *RLCK* genes in group I and also in group II; 27 in group III, and 13 in group IV. *RLCK* genes in group I had the low expression levels in the leaf, ear, and tassel. The log2 values were from −3 to 2. The genes in group II had high expression levels in root with log2 values from 2 to 6. The genes in group III showed a widely low expression levels in leaf, root, ear, and tassel. The genes in group IV showed a low expression levels in ear, tassel, and root, but relatively high levels in leaf. The expression profile showed that *RLCK* genes were widely expressed in maize, but there were some *RLCK* genes were specific expression in one tissue. For example, GRMZM2G15290, GRMZM2G473411, GRMZM2G090732, and GRMZM2G139223 had specific expression in root and GRMZM2G406601 and GRMZM2G055154 had specific expression in leaf. GRMZM2G091338 only had a relatively high expression in stele. GRMZM2G169080 and GRMZM2G177445 were only expressed in the base zones of leaf. GRMZM2G365319 and GRMZM2G073359 were expressed in the leaf zones of Base and −1 from ligule and root. GRMZM2G114899, GRMZM2G378547, GRMZM2G070961, and GRMZM2G301647 were only expressed in ear and tassel. The mentioned above indicated that these genes may have a specific function in the development processes of maize leaf, root, ear, and tassel.

## 3. Discussion

Overall, here we thought out analysis of genomic phylogeny, gene structure, chromosomal location, expansion mechanism of the maize, rice, and Arabidopsis RLCK superfamily. Previous research showed that the segmental duplication and the tandem duplication led to the expansion of RLKs in Arabidopsis and rice [13]. Our study results indicated that the segmental duplication might lead to the evolutionary differences of maize *RLCK* genes from Arabidopsis and rice. Gene duplication may be the main reason for the functional diversity on the gene level [20]. The maize *RLCK* genes distribution on chromosomes were near the ends of telomeres, indicating that a number of redundant or conservative sequences maybe existed in the positions which were far away from the centromere. The genes which were far away from the centromere may mutate more easier and thus produce the novel genes in the evolution [21]. The novel function may be generated by new genes to support survival and adapt for the changed environment.

The conservative kinase activity sites usually provide a stable environment for RLCKs to perform the function in cytoplasm. In our study, most of RLCK kinase activity sites were aspartic acid (d). The conservative kinase activity sites usually provide a stable environment for RLCKs to perform the functions in cytoplasm. In the phylogenetic tree, RLCK OS only contained the maize and rice *RLCK* genes, suggesting that RLCK OS may be a specific receptor like protein kinase of monocotyledon. It may provide strong evidence for the differentiation of monocotyledonous and dicotyledonous. The syntenic relationship between two species could provide an important basis for gene evolution [22]. Our research results further proved that the RLCKs among maize, rice, and Arabidopsis had a common ancestor, even orthologous in the evolutionary events [23]. The analysis of gene synteny and structure showed that some *RLCK* genes have appeared before the differentiation of monocotyledons and dicotyledons. Intron number might impact on the evolution in monocotyledons and dicotyledons, further influencing the diversity and complexity of *RLCK* genes structure during the long progress of evolution in maize [13]. 

It is important to understand *Ka/Ks* value for the evolution analysis. Generally, the genes which have undergone negative selection could add to the gene dosage [14]. The maize negative selection happened more strongly than other two species, suggesting that maize *RLCK* genes maybe have more chances to increase the gene dosage and generate the novel functions during evolution compared with Arabidopsis and rice. The earliest duplication time of *RLCK* genes in maize is 153.7 Mya which is prior to other two species, indicating that the maize had originated earlier than Arabidopsis and rice. Our results provide new insights into the evolution of RLCKs in maize, rice, and Arabidopsis, and will be useful in studies monocotyledonous and dicotyledonous development.

## 4. Materials and Methods

### 4.1. Identification and Protein Domain Prediction of RLCK Genes

In the present study, we chose RLKs from maize, rice, and Arabidopsis databases and relevant articles as base data sources. The related RLCK sequences were obtained from Phytozome (Available online: https://phytozome.jgi.doe.gov/pz/portal.html). The results were merged, and redundant sequences were deleted. For genes with more than one predicted isoforms, we analyzed only the longest peptide sequence of each gene. Online software PSORTb version 3.0.2 (Available online: http://www.psort.org/psortb/) was employed to predict sub-cellular localization using amino acid sequences of all obtained RLKs, and RLKs located in cytoplasm were selected to analyze kinase domains according to the SMART databases (http://smart.embl-heidelberg.de/smart/set_mode.cgi?NORMAL=1).

### 4.2. The Phylogram and Selection of RLCKs

All RLCK amino sequences in maize, rice, and Arabidopsis were aligned by ClustalX v1.83 [24] with default parameters. MEGA 5.0 [25] with NJ method was used to generate the phylogenetic tree. The *RLCK* gene intron information of maize, rice, and Arabidopsis were from the Ensembl Genomes (Available online: http://ensemblgenomes.org/).

The value of *Ka/Ks* was very important since it could estimate the changes of encoded amino acid sequences and gene functions, further estimate gene evolution. Each *Ka* and *Ks* value can be calculated by DnaSPv5.0 software (Available online: http://www.ub.edu/dnasp/). The gene duplication time (T) was calculated by the following equation [26]:

$${T=Ks/2\mu \times 10}^{-6}\;\mathrm{Mya}$$

where μ = 6.5 × 10^−9^ for maize and rice [27]; μ = 1.5 × 10^−8^ for Arabidopsis [28]; Software GenomePixelizer program (http://niblrrs.ucdavis.edu/GenomePixelizer/GenomePixelizer_Welcome.html) was employed to predict the duplication of *RLCK* genes in maize, rice, and Arabidopsis [29]. 

### 4.3. Distribution and Synteny of RLCK Genes

The starting positions on chromosome and synteny data of *RLCK* genes among maize, rice, and Arabidopsis were from Ensembl (Available online: http://www.ensembl.org/index.html). The chromosomal maps of *RLCK* genes in maize, rice, and Arabidopsis were visualized using MapInspect (http://www.plantbreeding.wur.nl/uk/software_mapinspect.html). 

### 4.4. Differential Expression of Maize RLCK Genes 

For analyzing the gene expression of *RLCK* genes in maize, the gene expression data were retrieved from Maize eFP Browser (http://bar.utoronto.ca/efp_maize/cgi-bin/efpWeb.cgi). The *RLCK* gene expression values were normalized by log2 values. Then the Heml software (http://hemi.biocuckoo.org/down.php) was employed to generate the maize RLCK gene expression profiles in hierarchial clustering.

## Supplementary Materials

Supplementary materials can be found at http://www.mdpi.com/1422-0067/19/11/3680/s1.
