# Supplementary material for: Evolution and Expression Characteristics of Receptor-Like Cytoplasmic Protein Kinases in Maize, Rice and Arabidopsis
Source: Int J Mol Sci. 2018 Nov 21;19(11):3680. doi: 10.3390/ijms19113680 (PMC6274858; doi:10.3390/ijms19113680)
Supplement: Supplementary file 1 [file ijms-19-03680-s001.zip › supplementary data/Table S1.docx]

Supplementary: Evolution and Expression Characteristics of Receptor-Like Cytoplasmic Protein Kinases in *Maize*, *Rice* and *Arabidopsis*

Mingxia Fan ^2,†^, Wenjuan Ma ^1,†^,·Chen Liu ^1^, Chunyu Zhang ^1^, Suwen Wu ^3^, Meiming Chen ^1^, Kuichen Liu^1^, Fengchun Cai ^1^ and Feng Lin ^1,^*

^1^ Biotechnology and Bioscience College, Shenyang Agricultural University, 120 Dongling Road, Shenyang 110866, China; 20152166@stu.syau.edu.cn (W.M.); liuchen@syau.edu.cn (C.L.); 1994500024@syau.edu.cn (C.Z); 20162164@stu.syau.edu.cn (M.C.); 2017220164@stu.syau.edu.cn (K.L.); 2017220160@stu.syau.edu.cn (F.C.)

^2^ Liaoning Key Laboratory of Urban Integrated Pest Management and Ecological Security, College of Life Science and Engineering Shenyang University, Shenyang 110044, China; syndlc@outlook.com

^3^ College of Science Institute, Shenyang Agricultural University No. 120 Dongling Road, Shenyang 110866, China; 2001500072@syau.edu.cn (S.W.)

***** Correspondence: fenglinsn@126.com; Tel.: +86-24-88487163

**†** These authors contributed equally to this work.

**Table S1.** The information of Gene ID, chromosomal location and intron numbers of RLCKs in maize. The related information of genes was searched from recent reports and Maize GDB in maize. The intron numbers were retrieved in Ensembl Genomes.

| Gene ID | Chromosome | Chromosomal location | Intron number |
| --- | --- | --- | --- |
| GRMZM2G355450 | 1 | 35020494-35022741 | 0 |
| GRMZM2G046848 | 1 | 89157835-89163331 | 8 |
| GRMZM2G153945 | 1 | 198655421-198659835 | 7 |
| AC217300.3_FG | 1 | 43615431-43621104 | 4 |
| GRMZM2G109566 | 1 | 7063949-7068108 | 4 |
| GRMZM2G125762 | 1 | 246673889-246679191 | 5 |
| GRMZM2G152901 | 1 | 240404326-240407505 | 3 |
| GRMZM2G175164 | 1 | 16842863-16848389 | 3 |
| GRMZM2G046297 | 1 | 63928070-63930264 | 2 |
| GRMZM2G101245 | 1 | 211229817-211232387 | 2 |
| GRMZM2G142390 | 1 | 72028758-72032846 | 2 |
| GRMZM2G068117 | 1 | 299188671-299193386 | 5 |
| GRMZM2G406601 | 1 | 15685183-15686574 | 1 |
| GRMZM2G087459 | 1 | 40787428-40790568 | 4 |
| GRMZM2G076423 | 1 | 12375052-12378601 | 5 |
| GRMZM2G119521 | 1 | 238750631-238752896 | 1 |
| GRMZM2G014366 | 1 | 5491457-5495322 | 6 |
| GRMZM2G176604 | 1 | 5491457-5495312 | 6 |
| GRMZM2G043470 | 1 | 30559326-30563850 | 4 |
| GRMZM2G181231 | 1 | 227366006-227368028 | 0 |
| GRMZM2G159992 | 1 | 110378380-110389175 | 6 |
| GRMZM2G026767 | 1 | 86770176-86776444 | 8 |
| GRMZM2G099598 | 1 | 7394883-7399605 | 8 |
| GRMZM2G169080 | 1 | 100085714-100090833 | 9 |
| GRMZM2G380227 | 1 | 200023869-200026523 | 5 |
| GRMZM2G177445 | 1 | 300057445-300062971 | 9 |
| GRMZM5G818431 | 1 | 32857576-32861916 | 6 |
| GRMZM2G114899 | 1 | 302328669-302332797 | 7 |
| GRMZM2G043799 | 1 | 10854114-10859532 | 5 |
| GRMZM2G055957 | 2 | 32540005-32544548 | 6 |
| GRMZM2G050861 | 2 | 53484383-53488254 | 6 |
| GRMZM2G007283 | 2 | 155301258-155306324 | 5 |
| GRMZM2G018059 | 2 | 50696434-50701184 | 6 |
| GRMZM2G471395 | 2 | 85504775-85506430 | 2 |
| GRMZM2G352281 | 2 | 94749230-94752775 | 4 |
| GRMZM2G349344 | 2 | 19414112-19420375 | 8 |
| GRMZM2G001668 | 2 | 174563828-174573555 | 6 |
| GRMZM2G015889 | 2 | 68587556-68592566 | 4 |
| GRMZM2G063533 | 2 | 202081304-202085828 | 3 |
| AC212835.3_FG | 2 | 22298801-22300512 | 3 |
| GRMZM2G051984 | 2 | 36102005-36106012 | 5 |
| GRMZM2G059740 | 2 | 4479443-4481492 | 1 |
| GRMZM2G076212 | 2 | 4431371-4433466 | 2 |
| GRMZM2G363066 | 2 | 4339356-4340894 | 1 |
| GRMZM2G150806 | 2 | 181872601-181877999 | 5 |
| GRMZM2G026301 | 2 | 163239922-163244823 | 7 |
| GRMZM2G168917 | 2 | 23112941-23114451 | 0 |
| GRMZM2G086577 | 2 | 4638970-4646084 | 5 |
| GRMZM2G142544 | 2 | 169619011-169623650 | 8 |
| GRMZM2G070961 | 3 | 224412744-224415725 | 5 |
| GRMZM2G161380 | 3 | 155956188-155960323 | 7 |
| GRMZM2G378547 | 3 | 58717043-58721522 | 7 |
| GRMZM2G346132 | 3 | 169649436-169653579 | 6 |
| GRMZM2G074381 | 3 | 37092813-37099056 | 4 |
| GRMZM2G335046 | 3 | 144476744-144477922 | 0 |
| GRMZM2G145709 | 3 | 155343255-155349107 | 6 |
| GRMZM2G091338 | 3 | 189536953-189538986 | 2 |
| GRMZM2G028037 | 3 | 225197920-225203269 | 6 |
| GRMZM2G034855 | 3 | 58480197-58484905 | 6 |
| GRMZM2G031400 | 3 | 214836511-214840264 | 7 |
| GRMZM2G068151 | 3 | 187651697-187655271 | 3 |
| GRMZM2G020915 | 4 | 186396699-186398119 | 0 |
| GRMZM2G140095 | 4 | 101589296-101597231 | 4 |
| GRMZM2G301513 | 4 | 235519093-235520318 | 1 |
| GRMZM2G301647 | 4 | 178304977-178308276 | 4 |
| GRMZM2G010488 | 4 | 157102240-157105817 | 5 |
| GRMZM2G105933 | 4 | 125336671-125339106 | 1 |
| GRMZM2G025127 | 4 | 245166373-245170503 | 5 |
| GRMZM2G042380 | 4 | 173977547-173982005 | 4 |
| GRMZM2G101687 | 4 | 154123890-154127016 | 2 |
| GRMZM2G073359 | 4 | 158063117-158065646 | 5 |
| GRMZM2G101754 | 4 | 173015140-173019293 | 6 |
| GRMZM2G099754 | 4 | 188525229-188530185 | 0 |
| GRMZM2G180775 | 4 | 153285690-153287274 | 1 |
| GRMZM2G163328 | 4 | 245923058-245925026 | 1 |
| GRMZM2G473411 | 4 | 173753513-173755232 | 2 |
| GRMZM2G125308 | 4 | 239886500-239888648 | 3 |
| GRMZM2G032337 | 4 | 212685378-212689960 | 10 |
| GRMZM2G048635 | 4 | 4785787-4789334 | 4 |
| GRMZM2G121715 | 5 | 2166748-2169144 | 5 |
| GRMZM2G159047 | 5 | 69615896-69617739 | 2 |
| GRMZM2G166719 | 5 | 22863385-22865343 | 1 |
| GRMZM2G169558 | 5 | 195387292-195388884 | 1 |
| GRMZM2G127984 | 5 | 179261732-179264249 | 7 |
| GRMZM2G137468 | 5 | 168785065-168791287 | 3 |
| GRMZM2G364172 | 5 | 221858646-221868145 | 3 |
| GRMZM2G158045 | 5 | 62838718-62841688 | 4 |
| GRMZM2G414899 | 5 | 59818499-59820642 | 2 |
| GRMZM2G055154 | 5 | 65523069-65526303 | 6 |
| GRMZM2G144042 | 5 | 2364265-2370543 | 4 |
| GRMZM2G110968 | 5 | 37785958-37788104 | 4 |
| GRMZM2G156824 | 5 | 72078215-72082309 | 5 |
| GRMZM2G166027 | 5 | 217590475-217592659 | 4 |
| GRMZM2G040964 | 5 | 175249948-175253741 | 6 |
| GRMZM2G037308 | 5 | 96005346-96009145 | 9 |
| GRMZM2G118939 | 5 | 99285143-99288586 | 3 |
| GRMZM2G121826 | 5 | 2173184-2178564 | 6 |
| GRMZM2G148962 | 5 | 1573730-1579082 | 7 |
| GRMZM2G061596 | 5 | 54404461-54406699 | 4 |
| GRMZM5G835629 | 6 | 141447130-141450518 | 3 |
| GRMZM2G381076 | 6 | 28516259-28520016 | 0 |
| GRMZM2G050701 | 6 | 96573519-96580085 | 7 |
| GRMZM2G146305 | 6 | 89229619-89232300 | 5 |
| AC234171.1_FG | 9 | 130019770-130021645 | 2 |
| GRMZM2G373435 | 6 | 112673176-112675478 | 3 |
| GRMZM2G010953 | 6 | 167702222-167706547 | 6 |
| GRMZM2G162702 | 6 | 138953327-138958838 | 2 |
| GRMZM2G171210 | 6 | 99390300-99393616 | 5 |
| GRMZM2G066432 | 6 | 156292403-156294916 | 4 |
| GRMZM2G133566 | 6 | 83034404-83037596 | 3 |
| GRMZM5G871928 | 6 | 31151359-31152962 | 4 |
| GRMZM2G471779 | 7 | 54089198-54093287 | 4 |
| GRMZM2G092550 | 7 | 109309083-109325401 | 8 |
| GRMZM2G029530 | 7 | 143640526-143645297 | 7 |
| GRMZM2G071877 | 7 | 39145451-39159208 | 8 |
| GRMZM2G048210 | 7 | 178728980-178733982 | 5 |
| GRMZM5G879570 | 7 | 148332449-148337057 | 4 |
| GRMZM2G004207 | 7 | 121815664-121817633 | 3 |
| GRMZM2G127031 | 7 | 170886190-170889860 | 3 |
| GRMZM2G132184 | 7 | 100359175-100364355 | 4 |
| GRMZM2G091919 | 7 | 147952084-147953976 | 3 |
| GRMZM2G351941 | 8 | 135478513-135481446 | 1 |
| GRMZM2G012966 | 8 | 143020563-143027781 | 9 |
| AC233953.1_FG | 8 | 164359286-164361050 | 6 |
| GRMZM2G343024 | 8 | 133390893-133396135 | 7 |
| GRMZM2G085038 | 8 | 271252-367057 | 4 |
| GRMZM2G140590 | 8 | 117765242-117779389 | 3 |
| GRMZM2G037585 | 8 | 164313713-164318382 | 4 |
| GRMZM2G139223 | 8 | 177053620-177055732 | 2 |
| GRMZM2G043069 | 8 | 124481690-124483802 | 4 |
| GRMZM2G168416 | 8 | 161465722-161467167 | 1 |
| GRMZM2G047588 | 8 | 137997924-138001492 | 3 |
| GRMZM2G147051 | 8 | 103220084-103223076 | 5 |
| GRMZM2G017386 | 8 | 150402835-150407178 | 7 |
| GRMZM2G090732 | 8 | 120111899-120114860 | 4 |
| GRMZM2G433433 | 9 | 20628842-20630538 | 2 |
| GRMZM2G305822 | 9 | 84217258-84220276 | 5 |
| GRMZM2G165433 | 9 | 11221616-11224738 | 3 |
| GRMZM2G009869 | 9 | 154764022-154768424 | 4 |
| GRMZM2G178787 | 9 | 154446529-154450626 | 7 |
| GRMZM2G157115 | 9 | 111942982-111948528 | 4 |
| AC149810.2_FG | 9 | 154782703-154785295 | 5 |
| GRMZM2G147373 | 9 | 130019770-130022099 | 2 |
| GRMZM2G055982 | 9 | 9516937-9519607 | 3 |
| GRMZM2G007801 | 9 | 15660933-15662836 | 2 |
| GRMZM2G317938 | 9 | 156831913-156836021 | 7 |
| GRMZM2G478876 | 9 | 144527869-144532705 | 5 |
| GRMZM2G431524 | 9 | 92553820-92557300 | 9 |
| GRMZM2G338376 | 9 | 111065404-111066906 | 2 |
| GRMZM2G104760 | 9 | 15924687-15927580 | 2 |
| GRMZM2G127050 | 9 | 154813696-154818452 | 7 |
| GRMZM2G054634 | 9 | 116754563-116759293 | 5 |
| GRMZM2G095302 | 9 | 111964172-111969046 | 8 |
| GRMZM2G365319 | 10 | 55246068-55249896 | 9 |
| GRMZM2G149943 | 10 | 48629503-48631730 | 0 |
| GRMZM2G061447 | 10 | 86464808-86469142 | 6 |
| GRMZM2G142832 | 10 | 139063732-139068557 | 5 |
| GRMZM2G007477 | 10 | 146944754-146946716 | 0 |
| GRMZM2G308365 | 10 | 146938274-146939628 | 0 |
| GRMZM2G481531 | 10 | 66206197-66207648 | 1 |
| GRMZM2G061537 | 10 | 129941495-129946469 | 5 |
| GRMZM2G418432 | 10 | 10863111-10866049 | 6 |
