# Supplementary material for: Evolution and Expression Characteristics of Receptor-Like Cytoplasmic Protein Kinases in Maize, Rice and Arabidopsis
Source: Int J Mol Sci. 2018 Nov 21;19(11):3680. doi: 10.3390/ijms19113680 (PMC6274858; doi:10.3390/ijms19113680)
Supplement: Supplementary file 1 [file ijms-19-03680-s001.zip › supplementary data/Table S2.docx]

Supplementary: Evolution and Expression Characteristics of Receptor-Like Cytoplasmic Protein Kinases in *Maize*, *Rice* and *Arabidopsis*

Mingxia Fan ^2,†^, Wenjuan Ma ^1,†^,·Chen Liu ^1^, Chunyu Zhang ^1^, Suwen Wu ^3^, Meiming Chen ^1^, Kuichen Liu^1^, Fengchun Cai ^1^ and Feng Lin ^1,^*

^1^ Biotechnology and Bioscience College, Shenyang Agricultural University, 120 Dongling Road, Shenyang 110866, China; 20152166@stu.syau.edu.cn (W.M.); liuchen@syau.edu.cn (C.L.); 1994500024@syau.edu.cn (C.Z); 20162164@stu.syau.edu.cn (M.C.); 2017220164@stu.syau.edu.cn (K.L.); 2017220160@stu.syau.edu.cn (F.C.)

^2^ Liaoning Key Laboratory of Urban Integrated Pest Management and Ecological Security, College of Life Science and Engineering Shenyang University, Shenyang 110044, China; syndlc@outlook.com

^3^ College of Science Institute, Shenyang Agricultural University No. 120 Dongling Road, Shenyang 110866, China; 2001500072@syau.edu.cn (S.W.)

***** Correspondence: fenglinsn@126.com; Tel.: +86-24-88487163

**†** These authors contributed equally to this work.

**Table S2.** The information of Gene ID, chromosomal location and intron numbers of RLCKs in *Arabidopsis* and rice. The related information of genes was searched from PlantP, TAIR and TGIR in *Arabidopsis* and rice. The intron numbers were retrieved in Ensembl Genomes.

| Gene ID | Chromosome | Location | Intron number |
| --- | --- | --- | --- |
| AT1G17540 | 1 | 6029277-6032754 | 10 |
| AT1G72760 | 1 | 27385421-27388274 | 10 |
| AT1G16760 | 1 | 5734234-5737307 | 8 |
| AT1G78940 | 1 | 29680822-29684161 | 8 |
| AT1G80870 | 1 | 30392133 - 30394383 | 0 |
| AT1G48210 | 1 | 17798431-17801987 | 7 |
| AT1G48220 | 1 | 17802863-17804955 | 6 |
| AT1G06700 | 1 | 2052481-2055548 | 7 |
| AT1G20650 | 1 | 7158244-7160526 | 5 |
| AT1G24030 | 1 | 8503234-8505460 | 6 |
| AT1G07870 | 1 | 2428773-2432057 | 6 |
| AT1G76370 | 1 | 28648449-28650484 | 5 |
| AT1G72540 | 1 | 27314932-27316669 | 4 |
| AT1G76360 | 1 | 28643038-28646712 | 5 |
| AT1G26970 | 1 | 9359682-9361833 | 4 |
| AT1G14370 | 1 | 4915576-4918138 | 5 |
| AT1G07570 | 1 | 2331170-2333738 | 5 |
| AT1G74490 | 1 | 27994700-27996742 | 4 |
| AT1G69790 | 1 | 26266609-26269013 | 4 |
| AT1G09440 | 1 | 3045513 - 3047893 | 7 |
| AT1G61740 | 1 | 22798069-22801073 | 3 |
| AT1G54820 | 1 | 20447316-20450861 | 4 |
| AT1G61590 | 1 | 22723501-22726074 | 4 |
| AT1G56720 | 1 | 21263420 - 21265620 | 8 |
| AT1G21010 | 1 | 7346156-7346973 | 0 |
| AT1G50990 | 1 | 18902930-18905204 | 6 |
| AT1G01740 | 1 | 272111-274569 | 9 |
| AT1G63500 | 1 | 23555686-23558899 | 9 |
| AT1G52540 | 1 | 19570058-19572204 | 3 |
| AT1G65190 | 1 | 24215644-24217328 | 0 |
| AT1G65250 | 1 | 24234338-24235808 | 0 |
| AT1G67470 | 1 | 25272182-25273576 | 0 |
| AT1G77280 | 1 | 29031468-29035882 | 10 |
| AT1G66460 | 1 | 24789735-24791988 | 4 |
| AT1G21590 | 1 | 7566232-7569902 | 9 |
| AT1G01540 | 1 | 195780-198684 | 6 |
| AT2G16750 | 2 | 7271012-7274693 | 10 |
| AT2G28930 | 2 | 12424303-12426674 | 5 |
| AT2G26290 | 2 | 11192137-11194259 | 4 |
| AT2G25220 | 2 | 1074271 -10745594 | 8 |
| AT2G28590 | 2 | 12249679-12251668 | 4 |
| AT2G30740 | 2 | 13095976-13098594 | 7 |
| AT2G43230 | 2 | 17966251-17968726 | 7 |
| AT2G45590 | 2 | 18786658-18788988 | 0 |
| AT2G07020 | 2 | 2908473-2911198 | 6 |
| AT2G19410 | 2 | 8404901-8409012 | 9 |
| AT2G24370 | 2 | 10369979-10373063 | 9 |
| AT2G41970 | 2 | 17520379-17522756 | 7 |
| AT2G39110 | 2 | 16319664-16321685 | 4 |
| AT2G28940 | 2 | 12426710-12428730 | 3 |
| AT2G17220 | 2 | 7487654-7490176 | 5 |
| AT2G02800 | 2 | 795515-799443 | 5 |
| AT2G45910 | 2 | 18894074-18898528 | 9 |
| AT2G05940 | 2 | 2287238-2289307 | 3 |
| AT2G07180 | 2 | 2980899-2983602 | 5 |
| AT2G39660 | 2 | 16531688-16533931 | 5 |
| AT2G20300 | 2 | 8755925-8760222 | 7 |
| AT2G42960 | 2 | 17868597-17870630 | 8 |
| AT2G28250 | 2 | 12043482-12046537 | 10 |
| AT2G11520 | 2 | 4618581-4621576 | 7 |
| AT2G30730 | 2 | 13093145-13094677 | 6 |
| AT2G18890 | 2 | 8183890-8186796 | 5 |
| AT3G01300 | 3 | 90598-93585 | 5 |
| AT3G17420 | 3 | 5959212-5962179 | 6 |
| AT3G58690 | 3 | 21709170-21711381 | 5 |
| AT3G09830 | 3 | 3016494-3018983 | 3 |
| AT3G49060 | 3 | 18186898-18191992 | 10 |
| AT3G20200 | 3 | 7047581-7051342 | 9 |
| AT3G26700 | 3 | 9809950-9812664 | 6 |
| AT3G52530 | 3 | 1948486-19485922 | 0 |
| AT3G57750 | 3 | 2139402 -21395288 | 0 |
| AT3G57700 | 3 | 21397542-21398853 | 0 |
| AT3G57710 | 3 | 21384676-21387399 | 0 |
| AT3G57720 | 3 | 21387722-21389726 | 0 |
| AT3G57730 | 3 | 21389964-21391562 | 0 |
| AT3G02810 | 3 | 608460-610985 | 4 |
| AT3G62220 | 3 | 23029100-23031155 | 7 |
| AT3G17410 | 3 | 5955909-5959106 | 7 |
| AT3G59350 | 3 | 21932525-21935192 | 5 |
| AT3G07070 | 3 | 2237958-2240074 | 5 |
| AT3G26940 | 3 | 9936582-9939269 | 4 |
| AT3G20530 | 3 | 7166047-7167929 | 4 |
| AT3G24790 | 3 | 9052982-9054531 | 4 |
| AT3G28690 | 3 | 10755039-10757868 | 4 |
| AT3G05140 | 3 | 1435698-1438341 | 6 |
| AT3G59110 | 3 | 21854719-21858204 | 7 |
| AT3G54030 | 3 | 20010927-20013693 | 8 |
| AT3G09240 | 3 | 2835668-2837956 | 9 |
| AT3G57640 | 3 | 21344800-21346057 | 0 |
| AT3G15890 | 3 | 5374248-5376275 | 3 |
| AT4G34500 | 4 | 16487754-16490954 | 6 |
| AT4G35230 | 4 | 16755112-16758091 | 8 |
| AT4G00710 | 4 | 290048-293453 | 10 |
| AT4G02630 | 4 | 1151613-1153356 | 0 |
| AT4G25160 | 4 | 12903191-12907338 | 9 |
| AT4G31230 | 4 | 15172897-15176109 | 8 |
| AT4G25390 | 4 | 12977341-12979568 | 0 |
| AT4G13190 | 4 | 7659435-7661106 | 3 |
| AT4G35600 | 4 | 16896202-16898876 | 5 |
| AT4G32000 | 4 | 15473971-15476685 | 9 |
| AT4G17660 | 4 | 9831359-9833006 | 3 |
| AT4G02010 | 4 | 881090-885399 | 7 |
| AT4G00330 | 4 | 142601-144523 | 6 |
| AT5G61560 | 5 | 24752850-24756718 | 10 |
| AT5G61550 | 5 | 24748102-24751871 | 9 |
| AT5G51270 | 5 | 20835137-20838262 | 9 |
| AT5G12000 | 5 | 3873906-3876914 | 6 |
| AT5G26146 | 5 | 9135913-9138264 | 2 |
| AT5G26150 | 5 | 9137461-9140099 | 6 |
| AT5G60080 | 5 | 24193181-24194909 | 4 |
| AT5G60090 | 5 | 24196082-24197725 | 4 |
| AT5G51770 | 5 | 21031030-21032994 | 0 |
| AT5G16500 | 5 | 5386675-5389165 | 4 |
| AT5G18610 | 5 | 6192443-6195753 | 4 |
| AT5G02800 | 5 | 635228-637503 | 4 |
| AT5G13160 | 5 | 4176579-4179885 | 4 |
| AT5G02290 | 5 | 470182-472784 | 6 |
| AT5G03320 | 5 | 802027-804398 | 4 |
| AT5G47070 | 5 | 19118543-19120909 | 3 |
| AT5G56460 | 5 | 22865110-22867996 | 5 |
| AT5G35380 | 5 | 13593429-13596293 | 8 |
| AT5G01020 | 5 | 5917-8444 | 4 |
| AT5G15080 | 5 | 4886114-4888788 | 5 |
| AT5G18500 | 5 | 6138383-6141630 | 8 |
| AT5G11020 | 5 | 3486242-3489234 | 10 |
| AT5G58940 | 5 | 23798562-23800823 | 6 |
| AT5G01060 | 5 | 22740-24847 | 6 |
| AT5G41260 | 5 | 16503774-16507475 | 9 |
| AT5G59010 | 5 | 23820368-23823265 | 9 |
| AT5G63940 | 5 | 25588075-25591458 | 9 |
| AT5G18910 | 5 | 6306828-6309419 | 8 |
| AT5G37790 | 5 | 15008172-15011152 | 5 |
| AT5G57670 | 5 | 23360400-23363757 | 9 |
| AT5G65530 | 5 | 26190592-26193106 | 7 |
| AT5G35960 | 5 | 14108524-14110536 | 6 |
| AT5G10520 | 5 | 3320370-3322910 | 7 |
| LOC_Os01g39970.1 | 1 | 22538988-22542228 | 4 |
| LOC_Os01g70970.1 | 1 | 41079469-41082009 | 9 |
| LOC_Os01g70410.2 | 1 | 40786166-40790612 | 9 |
| LOC_Os01g21970.1 | 1 | 12332795-12337227 | 7 |
| LOC_Os01g67340.2 | 1 | 39091728- 39095339 | 8 |
| LOC_Os01g15470.3 | 1 | 8682929-8687581 | 4 |
| LOC_Os01g71000.1 | 1 | 41095905-41100367 | 4 |
| LOC_Os01g57940.1 | 1 | 8682929-8687581 | 4 |
| LOC_Os01g74200.1 | 1 | 42981192-42985019 | 5 |
| LOC_Os01g40590.1 | 1 | 22918453-22923870 | 5 |
| LOC_Os01g21960.1 | 1 | 12326486-12331776 | 7 |
| LOC_Os01g47470.1 | 1 | 27130541-27134194 | 7 |
| LOC_Os01g44110.1 | 1 | 25281682-25286614 | 8 |
| LOC_Os01g41730.1 | 1 | 23624289-23627032 | 7 |
| LOC_Os01g63280.1 | 1 | 36683709-36687154 | 5 |
| LOC_Os01g60700.1 | 1 | 35096177-35100701 | 4 |
| LOC_Os01g20900.1 | 1 | 11677483-11685307 | 5 |
| LOC_Os02g12670.1 | 2 | 6616308-6623374 | 1 |
| LOC_Os02g05820.1 | 2 | 2869670-2874218 | 9 |
| LOC_Os02g12660.1 | 2 | 6609371-6614191 | 7 |
| LOC_Os02g54590.1 | 2 | 33434089-33439214 | 8 |
| LOC_Os02g35760.1 | 2 | 21474733-21477632 | 7 |
| LOC_Os02g30900.1 | 2 | 18446003-18450512 | 4 |
| LOC_Os02g02600.1 | 2 | 950728-954728 | 5 |
| LOC_Os02g57420.1 | 2 | 35190393-35194311 | 4 |
| LOC_Os02g43430.1 | 2 | 26208969-26213007 | 4 |
| LOC_Os02g44920.1 | 2 | 27215241-27217719 | 4 |
| LOC_Os02g57700.1 | 2 | 35334956-35340821 | 7 |
| LOC_Os02g58610.1 | 2 | 35821686-35822973 | 0 |
| LOC_Os02g53750.1 | 2 | 32915308-32918514 | 3 |
| LOC_Os02g34430.1 | 2 | 20622884-20626394 | 5 |
| LOC_Os02g08530.1 | 2 | 4604012-4607666 | 9 |
| LOC_Os02g02040.1 | 2 | 566043-572743 | 6 |
| LOC_Os02g43290.1 | 2 | 26090416-26092187 | 1 |
| LOC_Os02g01730.1 | 2 | 404245-406691 | 1 |
| LOC_Os02g57560.1 | 2 | 35263166-35265393 | 2 |
| LOC_Os02g09359.1 | 2 | 4808234-4817119 | 3 |
| LOC_Os03g02190.1 | 3 | 732486-734247 | 1 |
| LOC_Os03g29410.1 | 3 | 16749300-16754225 | 4 |
| LOC_Os03g01160.2 | 3 | 110086-114398 | 8 |
| LOC_Os03g31070.1 | 3 | 17702593-17710049 | 11 |
| LOC_Os03g31044.2 | 3 | 17680228-17688211 | 6 |
| LOC_Os03g31000.1 | 3 | 17660012-17667505 | 11 |
| LOC_Os03g14710.1 | 3 | 7998159-8000334 | 1 |
| LOC_Os03g51040.1 | 3 | 29180902-29183141 | 6 |
| LOC_Os03g62700.1 | 3 | 35480584-35486347 | 7 |
| LOC_Os03g12520.1 | 3 | 6626961-6630244 | 7 |
| LOC_Os03g17550.1 | 3 | 9751680-9755757 | 4 |
| LOC_Os03g03880.1 | 3 | 1765300-1770693 | 4 |
| LOC_Os03g07430.1 | 3 | 3767426-3771557 | 5 |
| LOC_Os03g08170.1 | 3 | 4173178-4179255 | 4 |
| LOC_Os03g03890.1 | 3 | 1772819-1775682 | 4 |
| LOC_Os03g60710.1 | 3 | 34502945-34508158 | 4 |
| LOC_Os03g24930.1 | 3 | 14206981-14209164 | 3 |
| LOC_Os03g06330.1 | 3 | 3165266-3168965 | 6 |
| LOC_Os03g16740.1 | 3 | 9269491-9273582 | 5 |
| LOC_Os03g03410.1 | 3 | 1457462-1461716 | 6 |
| LOC_Os03g12680.1 | 3 | 6762982-6767685 | 6 |
| LOC_Os03g61060.1 | 3 | 34688286-34690737 | 5 |
| LOC_Os03g05470.1 | 3 | 2707463-2710821 | 5 |
| LOC_Os03g04050.1 | 3 | 1852874-1857814 | 8 |
| LOC_Os03g13820.1 | 3 | 7482083-7487765 | 10 |
| LOC_Os03g62340.1 | 3 | 35312451-35317359 | 8 |
| LOC_Os04g32310.1 | 4 | 19365855-19371154 | 4 |
| LOC_Os04g30040.2 | 4 | 17929442-17940490 | 11 |
| LOC_Os04g30030.1 | 4 | 17913437-17916778 | 7 |
| LOC_Os04g39180.1 | 4 | 23294062-23298005 | 6 |
| LOC_Os04g45920.1 | 4 | 27194339-27197018 | 2 |
| LOC_Os04g47620.1 | 4 | 28262748-28265746 | 3 |
| LOC_Os04g41310.2 | 4 | 24512414-24515768 | 6 |
| LOC_Os04g56110.1 | 4 | 33418505-33421204 | 2 |
| LOC_Os04g56130.1 | 4 | 33426854-33428213 | 1 |
| LOC_Os04g56120.1 | 4 | 33421535-33423302 | 0 |
| LOC_Os04g35080.1 | 4 | 21323046-21326956 | 6 |
| LOC_Os04g45730.1 | 4 | 27058376-27060119 | 1 |
| LOC_Os04g56060.1 | 4 | 33386622-33391644 | 5 |
| LOC_Os05g36050.1 | 5 | 21342183-21345370 | 6 |
| LOC_Os05g30820.2 | 5 | 17885534-17888972 | 6 |
| LOC_Os05g04520.1 | 5 | 2096011-2100428 | 7 |
| LOC_Os05g03460.1 | 5 | 1461362-1463780 | 4 |
| LOC_Os05g30870.1 | 5 | 17922837-17930317 | 4 |
| LOC_Os05g41950.1 | 5 | 24569152-24571817 | 4 |
| LOC_Os05g02020.1 | 5 | 577144-580488 | 5 |
| LOC_Os05g33080.1 | 5 | 19398906-19403556 | 5 |
| LOC_Os05g25540.1 | 5 | 14840519-14844047 | 9 |
| LOC_Os05g38770.1 | 5 | 22745076-22747649 | 4 |
| LOC_Os06g04880.1 | 6 | 2140038-2146619 | 9 |
| LOC_Os06g37620.1 | 6 | 22271187- 22275542 | 8 |
| LOC_Os06g09230.2 | 6 | 4640591-4645101 | 4 |
| LOC_Os06g06760.1 | 6 | 3175775- 3181762 | 10 |
| LOC_Os06g05830.1 | 6 | 2660850-2663464 | 1 |
| LOC_Os06g50100.1 | 6 | 30346755-30350034 | 5 |
| LOC_Os06g45280.1 | 6 | 27363816-27369245 | 5 |
| LOC_Os06g48980.1 | 6 | 29675090-29678081 | 4 |
| LOC_Os06g51170.1 | 6 | 30953028-30956664 | 5 |
| LOC_Os06g10160.1 | 6 | 5196601-5199451 | 3 |
| LOC_Os06g13320.1 | 6 | 7325614-7327449 | 1 |
| LOC_Os06g07230.1 | 6 | 3460178-3465373 | 6 |
| LOC_Os06g44430.1 | 6 | 26833860-26836084 | 3 |
| LOC_Os06g07070.1 | 6 | 3360524-3363523 | 3 |
| LOC_Os06g45350.1 | 6 | 27412678-27417601 | 8 |
| LOC_Os06g47820.1 | 6 | 28941271-28943704 | 5 |
| LOC_Os07g04810.1 | 7 | 2128418-2130049 | 1 |
| LOC_Os07g04820.1 | 7 | 2131760-2133500 | 1 |
| LOC_Os07g12480.1 | 7 | 7110757-7112739 | 0 |
| LOC_Os07g49470.1 | 7 | 29630560-29633467 | 4 |
| LOC_Os07g47270.1 | 7 | 28250042-28253666 | 4 |
| LOC_Os07g48730.1 | 7 | 29179661-29181610 | 3 |
| LOC_Os07g31290.1 | 7 | 18528508-18531797 | 6 |
| LOC_Os07g42200.1 | 7 | 25255060-25260015 | 3 |
| LOC_Os08g28710.1 | 8 | 17558062-17560192 | 1 |
| LOC_Os08g35600.1 | 8 | 22415787-22418614 | 3 |
| LOC_Os08g18920.1 | 8 | 11274669-11279209 | 9 |
| LOC_Os08g15060.1 | 8 | 9101238-9105746 | 10 |
| LOC_Os09g39650.1 | 9 | 22753304-22757508 | 11 |
| LOC_Os09g33860.1 | 9 | 19991623-19995868 | 8 |
| LOC_Os09g39930.1 | 9 | 22902939-22904835 | 1 |
| LOC_Os09g20880.2 | 9 | 12565502-12569202 | 6 |
| LOC_Os09g36320.1 | 9 | 20962030-20967594 | 3 |
| LOC_Os09g39640.1 | 9 | 22747357-22752211 | 2 |
| LOC_Os09g39620.1 | 9 | 22736559-22741162 | 9 |
| LOC_Os09g27010.1 | 9 | 16422278-16425218 | 3 |
| LOC_Os09g19700.1 | 9 | 11789182-11793501 | 5 |
| LOC_Os09g03620.1 | 9 | 1803961-1810819 | 6 |
| LOC_Os10g41220.1 | 10 | 22136928-22141736 | 9 |
| LOC_Os10g40100.2 | 10 | 21467608-21472768 | 11 |
| LOC_Os10g40060.1 | 10 | 21454034-21459779 | 8 |
| LOC_Os10g12620.1 | 10 | 7022929-7031173 | 9 |
| LOC_Os10g26520.1 | 10 | 13823476-13829535 | 5 |
| LOC_Os10g30600.1 | 10 | 15920835-15923997 | 4 |
| LOC_Os10g25550.1 | 10 | 13219950-13226158 | 4 |
| LOC_Os10g29620.1 | 10 | 15402600-15405854 | 5 |
| LOC_Os10g38920.1 | 10 | 20731150-20734054 | 6 |
| LOC_Os10g35450.1 | 10 | 18970490-18975230 | 7 |
| LOC_Os10g39670.1 | 10 | 21199763-21204782 | 8 |
| LOC_Os10g42110.1 | 10 | 22650009-22654267 | 9 |
| LOC_Os10g01060.1 | 10 | 67119-72971 | 8 |
| LOC_Os10g37190.1 | 10 | 19899083-19900928 | 1 |
| LOC_Os11g11780.1 | 11 | 6539074-6541690 | 6 |
| LOC_Os11g11890.1 | 11 | 6596374-6603010 | 9 |
| LOC_Os11g39420.1 | 11 | 23459658-23475516 | 15 |
| LOC_Os11g10640.1 | 11 | 5835229-5841560 | 4 |
| LOC_Os11g10710.1 | 11 | 5873795-5880713 | 14 |
| LOC_Os11g39530.1 | 11 | 23537272-23545497 | 6 |
| LOC_Os11g39490.1 | 11 | 23508365-23513127 | 4 |
| LOC_Os11g39450.1 | 11 | 23488660-23496017 | 7 |
| LOC_Os11g17380.1 | 11 | 9688431-9695220 | 5 |
| LOC_Os11g44550.1 | 11 | 26934169-26935437 | 1 |
| LOC_Os11g44250.1 | 11 | 26741918-26743907 | 1 |
| LOC_Os11g44260.1 | 11 | 26750902-26752365 | 2 |
| LOC_Os11g44430.1 | 11 | 26877874-26880047 | 1 |
| LOC_Os11g25510.1 | 11 | 14525685-14527210 | 0 |
| LOC_Os11g01740.1 | 11 | 397418-401946 | 8 |
| LOC_Os11g40380.1 | 11 | 24071594-24075621 | 9 |
| LOC_Os11g40430.1 | 11 | 24101932-24106267 | 3 |
| LOC_Os11g08950.1 | 11 | 4735257-4738797 | 10 |
| LOC_Os11g42440.1 | 11 | 25542740-25547535 | 4 |
| LOC_Os12g01740.1 | 12 | 439034-443732 | 9 |
| LOC_Os12g30180.1 | 12 | 18115908-18120680 | 6 |
| LOC_Os12g31560.1 | 12 | 18984634 -18986648 | 2 |
