# Supplementary material for: Evolution and Expression Characteristics of Receptor-Like Cytoplasmic Protein Kinases in Maize, Rice and Arabidopsis
Source: Int J Mol Sci. 2018 Nov 21;19(11):3680. doi: 10.3390/ijms19113680 (PMC6274858; doi:10.3390/ijms19113680)
Supplement: Supplementary file 1 [file ijms-19-03680-s001.zip › supplementary data/Table S3.docx]

Supplementary: Evolution and Expression Characteristics of Receptor-Like Cytoplasmic Protein Kinases in *Maize*, *Rice* and *Arabidopsis*

Mingxia Fan ^2,†^, Wenjuan Ma ^1,†^,·Chen Liu ^1^, Chunyu Zhang ^1^, Suwen Wu ^3^, Meiming Chen ^1^, Kuichen Liu^1^, Fengchun Cai ^1^ and Feng Lin ^1,^*

^1^ Biotechnology and Bioscience College, Shenyang Agricultural University, 120 Dongling Road, Shenyang 110866, China; 20152166@stu.syau.edu.cn (W.M.); liuchen@syau.edu.cn (C.L.); 1994500024@syau.edu.cn (C.Z); 20162164@stu.syau.edu.cn (M.C.); 2017220164@stu.syau.edu.cn (K.L.); 2017220160@stu.syau.edu.cn (F.C.)

^2^ Liaoning Key Laboratory of Urban Integrated Pest Management and Ecological Security, College of Life Science and Engineering Shenyang University, Shenyang 110044, China; syndlc@outlook.com

^3^ College of Science Institute, Shenyang Agricultural University No. 120 Dongling Road, Shenyang 110866, China; 2001500072@syau.edu.cn (S.W.)

***** Correspondence: fenglinsn@126.com; Tel.: +86-24-88487163

**†** These authors contributed equally to this work.

**Table S3.** The log2 values of 90 detected maize RLCK genes in the differently development stages of three tissues.

|  | V16_floret; top ear (5 cm) floret | V1_leaf; 1st and 2nd leaf | 10DAP_leaf; 15 cm tip of 2nd leaf above top ear | R1_husk; most inner husk of top ear | VE_leaf; coleoptile | V2_seminal_root; seminal root | V16_silk; top ear (5 cm) silk | V8_V9_tassel; tassel 12-14 cm | 24DAP_root; nodal root | R1_ovule; R1-ovule of top ear | 5DAP_cob; cob of top ear | V7_tassel; tassel 2 cm | V5_seminal_root; seminal root | V15_tassel; spikelet of tassel (~22 cm) | 24DAP_pericarp; pericarp of top ear | V10_ear; top ear 1-1.5 cm | V5_leaf; eighth leaf, 15 cm including tip | 10DAP_endosperm; endosperm of top ear | 31DAP_leaf; 15 cm tip of 2nd leaf above top ear | R1_silk; silk of top ear | V15_ear; top ear 3-3.5 cm | 17DAP_embryo; embryo of top ear | V5_nodal_root; nodal root | R1_root; adult nodal root | V1_root; seminal root |
| --- | --- | --- | --- | --- | --- | --- | --- | --- | --- | --- | --- | --- | --- | --- | --- | --- | --- | --- | --- | --- | --- | --- | --- | --- | --- |
| GRMZM2G001668 | 7.202026 | 7.643784 | 7.815255 | 6.604516 | 7.123914 | 6.952101 | 7.296824 | 7.502872 | 6.241268 | 6.760088 | 6.883743 | 7.467443 | 7.030115 | 7.125672 | 7.931506 | 7.535742 | 8.085393 | 9.054333 | 7.468991 | 7.68818 | 7.383186 | 7.545273 | 7.106955 | 6.143842 | 7.066089 |
| GRMZM2G007283 | 9.746985 | 8.678001 | 7.923149 | 9.632668 | 9.107662 | 8.682327 | 9.485507 | 9.610527 | 8.478244 | 9.882597 | 9.796218 | 9.847543 | 8.917521 | 9.803162 | 9.548552 | 9.670709 | 8.268565 | 9.719799 | 7.692162 | 10.25014 | 9.703661 | 9.242603 | 9.085871 | 9.686693 | 8.655459 |
| GRMZM2G007477 | 6.853372 | 7.553898 | 8.214513 | 6.864929 | 7.051372 | 8.395234 | 6.929436 | 6.947082 | 9.136965 | 6.699746 | 6.706669 | 6.680324 | 8.53815 | 7.791749 | 6.989934 | 6.897361 | 8.075426 | 6.983906 | 8.411087 | 7.841659 | 6.819285 | 6.425426 | 8.284523 | 8.361066 | 8.114419 |
| GRMZM2G010953 | 10.97865 | 8.458489 | 7.32364 | 9.839818 | 9.781573 | 9.178889 | 10.99818 | 10.19274 | 8.702311 | 10.65261 | 10.10027 | 10.57035 | 9.333513 | 9.188935 | 10.21862 | 10.85324 | 7.599318 | 10.80913 | 6.863195 | 10.26087 | 10.91114 | 10.2346 | 9.606054 | 9.378685 | 9.197905 |
| GRMZM2G012966 | 4.703211 | 10.13245 | 10.2915 | 7.569552 | 7.667183 | 4.650765 | 6.226894 | 7.3474 | 4.289834 | 6.782671 | 7.867341 | 6.152995 | 4.707635 | 8.360276 | 4.957915 | 5.463688 | 10.57732 | 4.3875 | 10.31774 | 8.40412 | 6.01859 | 3.894333 | 4.104337 | 4.142413 | 4.456149 |
| GRMZM2G014366 | 8.46573 | 9.426852 | 7.082575 | 8.830135 | 8.886001 | 8.418106 | 8.712355 | 9.261413 | 7.197217 | 9.684714 | 9.135453 | 8.754253 | 8.392017 | 7.242984 | 8.819445 | 8.571411 | 7.941224 | 8.320349 | 7.137401 | 8.877928 | 8.445015 | 9.157928 | 8.734439 | 8.214513 | 8.455286 |
| GRMZM2G015889 | 7.928311 | 7.645875 | 7.156639 | 8.753083 | 7.775182 | 7.504064 | 8.156842 | 8.234291 | 6.537296 | 8.046306 | 8.298017 | 7.890143 | 7.505732 | 7.839456 | 8.147001 | 8.009885 | 7.60844 | 7.907131 | 6.938991 | 8.957944 | 7.90665 | 8.462993 | 7.770631 | 8.163599 | 7.559109 |
| GRMZM2G017386 | 8.02259 | 6.88533 | 5.586164 | 7.411511 | 7.768912 | 6.703765 | 8.249208 | 8.076816 | 6.167318 | 7.70501 | 7.110823 | 8.17732 | 6.453847 | 6.070604 | 6.978882 | 7.289097 | 6.239551 | 7.838195 | 5.904243 | 7.68629 | 7.731591 | 7.523719 | 6.933691 | 6.207502 | 7.087038 |
| GRMZM2G018059 | 6.860839 | 7.006635 | 7.02048 | 7.002252 | 7.023255 | 7.206428 | 7.103288 | 6.778603 | 7.027795 | 6.971429 | 6.806582 | 6.727376 | 7.116032 | 7.02381 | 7.152183 | 6.748059 | 7.378078 | 7.068671 | 7.081936 | 7.10507 | 6.826676 | 6.984703 | 7.25833 | 6.975217 | 7.362382 |
| GRMZM2G020915 | 4.048759 | 4.086614 | 3.939227 | 4.19456 | 4.131754 | 4.365273 | 4.121844 | 3.874797 | 4.095924 | 4.025915 | 3.943921 | 3.892391 | 4.253233 | 7.61191 | 4.275752 | 3.878725 | 4.189825 | 4.304511 | 4.06264 | 4.221104 | 4.014355 | 3.99458 | 4.163499 | 4.157044 | 4.275752 |
| GRMZM2G026301 | 9.644361 | 6.127221 | 6.647602 | 6.678072 | 7.680184 | 6.068026 | 8.699191 | 7.643856 | 5.752749 | 8.658104 | 7.057342 | 9.487297 | 6.124121 | 6.245838 | 6.998872 | 9.934118 | 6.297375 | 8.176423 | 6.690836 | 6.94357 | 9.779982 | 9.149595 | 6.496974 | 6.102658 | 6.148731 |
| GRMZM2G026767 | 7.810764 | 7.898813 | 9.352595 | 8.112075 | 7.150763 | 8.438251 | 7.266037 | 7.931447 | 9.569115 | 8.513372 | 8.639666 | 8.028624 | 8.983792 | 9.063287 | 8.575048 | 8.047069 | 8.411469 | 7.31904 | 9.29687 | 8.25979 | 7.956986 | 7.515148 | 8.53582 | 9.228987 | 8.302182 |
| GRMZM2G028037 | 8.907912 | 7.533096 | 7.304055 | 7.994523 | 8.304922 | 7.895908 | 8.85823 | 8.204816 | 8.585714 | 8.568146 | 8.692337 | 9.050991 | 8.223616 | 7.821136 | 7.333513 | 9.258118 | 7.079805 | 7.162895 | 7.480346 | 7.779391 | 9.209843 | 8.226942 | 7.968609 | 8.143332 | 8.042371 |
| GRMZM2G029530 | 10.2427 | 9.7829 | 10.20983 | 10.19779 | 9.705442 | 10.32216 | 10.96049 | 11.04562 | 10.28951 | 10.42165 | 10.1065 | 10.4552 | 10.2092 | 9.679023 | 11.08293 | 9.908573 | 10.11442 | 11.29874 | 10.13686 | 11.24556 | 10.20222 | 11.0729 | 10.21653 | 9.691569 | 10.43058 |
| GRMZM2G031400 | 6.783064 | 6.804131 | 6.12784 | 7.089689 | 6.79818 | 6.728601 | 7.5216 | 7.136376 | 6.184875 | 6.63125 | 7.254178 | 6.809929 | 6.381802 | 6.169123 | 6.552439 | 6.781753 | 6.381283 | 6.103078 | 6.325171 | 6.814294 | 6.890811 | 6.85412 | 6.751142 | 6.173727 | 7.204278 |
| GRMZM2G032337 | 6.560868 | 5.705149 | 5.596637 | 6.216067 | 6.320485 | 5.938286 | 6.781229 | 6.430954 | 5.453518 | 6.217231 | 6.074034 | 6.4707 | 5.729553 | 7.041112 | 5.653633 | 6.687481 | 5.587965 | 5.847496 | 5.489607 | 5.643856 | 6.771225 | 6.792205 | 6.154616 | 5.49857 | 6.318859 |
| GRMZM2G034855 | 8.748662 | 8.323415 | 5.507795 | 10.14229 | 8.513925 | 9.283436 | 9.213712 | 9.43227 | 8.574631 | 8.898087 | 9.569419 | 8.750104 | 9.609548 | 8.620073 | 8.875227 | 8.19175 | 6.391287 | 8.808803 | 5.390943 | 9.055608 | 8.587402 | 8.351116 | 10.02721 | 10.34357 | 9.450221 |
| GRMZM2G037308 | 8.65571 | 7.195544 | 5.526069 | 9.963517 | 7.463688 | 7.501598 | 7.01614 | 8.619156 | 6.05745 | 8.128458 | 9.284662 | 7.805292 | 7.157448 | 7.997349 | 7.619193 | 9.206502 | 6.41312 | 6.491372 | 5.710118 | 7.01692 | 8.858323 | 8.14634 | 7.893059 | 6.970739 | 7.718088 |
| GRMZM2G037585 | 7.546123 | 7.565521 | 7.263786 | 7.752481 | 7.62432 | 7.539934 | 7.836997 | 7.668247 | 7.897966 | 7.793116 | 7.737552 | 7.822666 | 7.730029 | 7.60021 | 8.152843 | 7.897845 | 7.465566 | 7.633141 | 7.332439 | 7.833776 | 7.645154 | 7.582556 | 7.515463 | 7.682433 | 7.444435 |
| GRMZM2G040964 | 8.120445 | 6.749802 | 4.636335 | 7.823622 | 7.205647 | 8.00214 | 7.75709 | 7.889413 | 5.514753 | 8.11197 | 8.492734 | 7.48301 | 7.883987 | 6.671718 | 8.520854 | 7.670161 | 4.778734 | 8.434169 | 5.09043 | 7.204473 | 7.792074 | 6.899417 | 8.094447 | 8.530055 | 8.186263 |
| GRMZM2G042380 | 5.524816 | 5.145677 | 4.922674 | 5.531069 | 5.277241 | 5.866166 | 5.001352 | 5.552131 | 6.41515 | 5.467606 | 5.44427 | 5.805808 | 5.920532 | 5.68173 | 6.682995 | 5.793636 | 5.348374 | 6.19022 | 5.289466 | 5.477677 | 5.51885 | 6.035184 | 5.841218 | 5.953032 | 5.961855 |
| GRMZM2G043069 | 4.825786 | 4.960697 | 5.004052 | 4.838448 | 4.735522 | 4.769243 | 4.933573 | 4.732269 | 4.751678 | 4.770829 | 4.604664 | 4.62527 | 4.676944 | 8.495335 | 5.19101 | 4.794936 | 5.472163 | 5.116864 | 4.929318 | 4.798569 | 4.823749 | 4.814038 | 4.730096 | 4.857483 | 4.798051 |
| GRMZM2G043799 | 8.856675 | 7.366497 | 9.146747 | 8.393047 | 7.281513 | 9.800042 | 8.645334 | 8.56354 | 10.87632 | 9.316983 | 8.348418 | 8.026191 | 10.39969 | 8.851187 | 7.560791 | 7.821072 | 8.172277 | 7.194954 | 9.117799 | 9.651931 | 7.853122 | 6.431957 | 9.503826 | 10.34819 | 9.207844 |
| GRMZM2G047588 | 6.027464 | 6.330917 | 6.006971 | 6.478648 | 6.107479 | 6.782278 | 6.10957 | 6.33396 | 7.354293 | 6.0917 | 6.40446 | 6.134837 | 6.637204 | 6.435795 | 5.936402 | 6.183685 | 6.423578 | 6.366147 | 6.161081 | 6.517906 | 6.193575 | 6.037163 | 6.509854 | 6.821072 | 6.672991 |
| GRMZM2G048210 | 6.448405 | 6.399342 | 6.590812 | 6.447414 | 6.18428 | 7.001014 | 6.148324 | 6.379725 | 7.270436 | 6.700717 | 6.51822 | 6.638943 | 7.010892 | 6.447744 | 6.26059 | 6.623369 | 6.528728 | 5.759688 | 6.549053 | 6.555049 | 6.511911 | 6.284662 | 6.839708 | 6.829976 | 7.096557 |
| GRMZM2G051984 | 5.367721 | 7.775577 | 10.24617 | 5.985728 | 5.631104 | 6.292414 | 5.516646 | 5.9855 | 7.2751 | 5.701826 | 5.793636 | 4.95977 | 6.492494 | 7.576598 | 5.515069 | 5.441284 | 9.1698 | 6.020591 | 9.878189 | 5.399855 | 5.319401 | 5.23764 | 6.669594 | 8.457955 | 6.998196 |
| GRMZM2G054634 | 11.07067 | 6.893605 | 6.253233 | 8.582707 | 9.106511 | 7.844674 | 10.07251 | 9.116708 | 6.66249 | 9.97298 | 8.799055 | 10.50828 | 7.971946 | 7.050175 | 8.112492 | 11.75935 | 6.8098 | 9.145448 | 6.488161 | 8.508468 | 11.16299 | 9.566016 | 8.664696 | 7.652128 | 8.060426 |
| GRMZM2G055957 | 7.461398 | 6.943101 | 6.917074 | 7.383445 | 7.194067 | 8.764241 | 7.670869 | 7.777222 | 8.743892 | 7.506367 | 8.816088 | 8.392661 | 8.633504 | 7.114679 | 7.992882 | 8.178565 | 7.180605 | 8.235105 | 6.901832 | 8.100137 | 7.70335 | 7.358256 | 8.50315 | 8.977394 | 8.562853 |
| GRMZM2G059740 | 5.285032 | 5.438958 | 6.258896 | 5.177519 | 5.330558 | 7.790381 | 5.301953 | 5.021035 | 8.92843 | 5.004951 | 5.223036 | 5.098874 | 8.414854 | 6.432458 | 5.26341 | 5.19928 | 5.608514 | 4.861459 | 6.107269 | 7.174227 | 5.119356 | 4.864929 | 7.087887 | 8.482082 | 7.106327 |
| GRMZM2G061447 | 11.1781 | 10.45989 | 11.38262 | 11.3679 | 10.45422 | 11.25251 | 11.11012 | 11.14696 | 11.15869 | 11.27816 | 11.57076 | 10.9127 | 11.33403 | 10.55527 | 10.76883 | 11.02051 | 10.1579 | 9.497133 | 10.21728 | 11.54607 | 11.13844 | 10.21428 | 11.33918 | 11.71943 | 11.33355 |
| GRMZM2G063897 | 7.345804 | 7.326699 | 6.05745 | 7.464423 | 7.483251 | 7.257011 | 7.104651 | 7.303872 | 6.128046 | 7.272397 | 7.248117 | 7.322289 | 7.235057 | 6.286512 | 5.997292 | 7.268565 | 6.530289 | 6.658783 | 6.018812 | 6.243174 | 7.106537 | 6.805421 | 7.176323 | 7.466138 | 7.746313 |
| GRMZM2G066432 | 5.626731 | 5.218781 | 5.155021 | 5.441284 | 5.024586 | 5.593951 | 8.351293 | 4.790772 | 4.93121 | 5.078097 | 4.900142 | 4.802193 | 5.290203 | 9.514142 | 5.209453 | 4.753818 | 5.525443 | 4.892391 | 5.123914 | 7.853684 | 4.844486 | 5.122673 | 5.372952 | 5.2791 | 5.846243 |
| GRMZM2G068117 | 8.683556 | 7.522228 | 8.220717 | 7.818774 | 7.778669 | 8.108002 | 8.265334 | 8.546431 | 8.610619 | 8.319446 | 8.196627 | 8.670975 | 8.3332 | 8.586877 | 9.263316 | 8.87992 | 7.881665 | 9.530543 | 8.24103 | 8.452282 | 8.70936 | 8.884964 | 8.18651 | 8.374996 | 7.843356 |
| GRMZM2G068151 | 8.860994 | 7.434878 | 7.958727 | 9.097453 | 8.336864 | 8.740725 | 8.544733 | 9.160224 | 9.176173 | 9.325305 | 9.728329 | 8.777222 | 8.689159 | 7.847872 | 8.213007 | 8.88996 | 7.427606 | 7.578788 | 8.118733 | 8.387587 | 8.789729 | 7.754754 | 8.46021 | 8.414897 | 8.403268 |
| GRMZM2G076212 | 4.232661 | 5.501758 | 6.867896 | 7.455574 | 4.970394 | 7.604664 | 4.354029 | 4.782933 | 9.286604 | 4.32337 | 6.570311 | 4.093391 | 8.529079 | 7.379812 | 5.735793 | 4.78398 | 5.934988 | 4.382667 | 6.319762 | 6.728601 | 3.971773 | 4.297191 | 8.777683 | 9.065524 | 7.943218 |
| GRMZM2G076423 | 6.988117 | 8.989877 | 9.219653 | 7.77538 | 7.856861 | 10.34803 | 6.85997 | 7.980882 | 11.23633 | 7.632632 | 8.627096 | 7.937874 | 10.67479 | 9.430473 | 5.665336 | 8.076816 | 9.311135 | 5.000901 | 9.611375 | 8.471716 | 7.932038 | 7.138937 | 9.634049 | 10.18196 | 9.560963 |
| GRMZM2G085038 | 9.658104 | 7.920532 | 9.439062 | 9.354844 | 8.508151 | 9.177469 | 8.642413 | 9.448571 | 9.557157 | 9.68853 | 9.842932 | 9.775166 | 9.439021 | 9.123061 | 8.348064 | 9.899493 | 8.415784 | 8.326429 | 9.174751 | 8.889565 | 9.858618 | 9.459186 | 9.426013 | 9.796883 | 9.054034 |
| GRMZM2G086577 | 6.151575 | 6.681309 | 6.432291 | 6.320485 | 6.194363 | 6.157448 | 6.203984 | 6.185867 | 6.127633 | 6.301039 | 6.054414 | 6.02148 | 6.160275 | 6.116656 | 5.956754 | 6.094658 | 6.985956 | 6.16792 | 6.527477 | 6.060047 | 5.999098 | 6.123501 | 6.150153 | 5.956289 | 6.171727 |
| GRMZM2G087459 | 9.342364 | 8.501041 | 9.129103 | 9.653812 | 9.069369 | 8.606516 | 9.396455 | 9.148426 | 10.19328 | 9.840746 | 9.8384 | 9.93251 | 9.101293 | 8.488121 | 8.778241 | 9.437731 | 8.34203 | 5.655638 | 9.008793 | 10.21284 | 9.184206 | 8.390255 | 8.908393 | 9.7029 | 8.550631 |
| GRMZM2G091338 | 3.783457 | 4.266787 | 4.065228 | 4.975447 | 4.082362 | 4.269781 | 4.126808 | 3.844988 | 3.760221 | 3.971773 | 3.819668 | 3.767655 | 3.917432 | 9.561689 | 4.090007 | 3.790772 | 4.116032 | 4.108524 | 3.88655 | 3.917432 | 3.831877 | 3.901108 | 3.989139 | 4.055716 | 4.109361 |
| GRMZM2G092550 | 6.196135 | 5.479619 | 5.024586 | 5.207502 | 5.450551 | 5.225738 | 5.96786 | 5.601399 | 5.142005 | 5.796494 | 5.389567 | 5.66846 | 5.427606 | 5.063503 | 5.346248 | 6.193772 | 5.468909 | 5.111031 | 5.127633 | 5.769507 | 5.909773 | 5.633722 | 5.356144 | 5.264536 | 5.314697 |
| GRMZM2G092776 | 7.090007 | 5.953265 | 6.922079 | 6.91576 | 6.513649 | 7.415742 | 6.790642 | 7.139858 | 7.56354 | 7.104861 | 7.207405 | 7.143128 | 7.334229 | 6.881787 | 7.708808 | 7.393777 | 6.413628 | 7.442861 | 6.896272 | 6.950352 | 7.138733 | 7.609622 | 7.273423 | 7.566739 | 7.248117 |
| GRMZM2G095302 | 7.202222 | 8.095292 | 8.493935 | 6.813781 | 6.172127 | 7.077457 | 7.137606 | 7.377558 | 8.244554 | 6.506526 | 6.545968 | 7.2791 | 7.314153 | 7.74032 | 5.486071 | 7.776236 | 8.570539 | 5.480265 | 8.738667 | 7.434295 | 7.383359 | 6.915043 | 7.006522 | 7.053111 | 7.266693 |
| GRMZM2G101245 | 5.93546 | 5.815063 | 5.90641 | 6.185867 | 5.951168 | 6.201046 | 5.840211 | 5.661065 | 5.997067 | 5.848998 | 6.425426 | 5.655924 | 6.077029 | 5.726286 | 5.852498 | 5.950235 | 6.055065 | 5.788686 | 5.871351 | 5.982537 | 5.964861 | 5.503031 | 6.176522 | 6.914923 | 6.338246 |
| GRMZM2G104760 | 6.651626 | 7.817751 | 8.955824 | 7.73843 | 7.098559 | 8.584662 | 7.375908 | 7.759489 | 9.804196 | 7.377905 | 8.219169 | 6.76633 | 8.691883 | 8.625197 | 7.971888 | 6.690417 | 8.565331 | 7.620073 | 9.148934 | 8.615335 | 6.699052 | 6.824641 | 8.338112 | 8.693069 | 8.238644 |
| GRMZM2G110412 | 4.82273 | 6.572738 | 5.017477 | 6.651626 | 5.54566 | 6.142005 | 4.944858 | 5.632559 | 5.079805 | 4.927422 | 6.281513 | 4.638074 | 5.974988 | 5.216455 | 5.854494 | 4.877744 | 5.864681 | 4.958843 | 5.175125 | 5.109361 | 4.839456 | 4.615299 | 6.305788 | 6.695994 | 6.882032 |
| GRMZM2G110968 | 6.17732 | 6.238596 | 6.882399 | 6.843984 | 6.190022 | 7.732473 | 6.541484 | 6.072535 | 8.204571 | 6.635464 | 6.651052 | 6.033203 | 7.485829 | 6.629211 | 6.923506 | 6.225738 | 6.479295 | 6.080871 | 6.691953 | 7.47622 | 5.998647 | 5.955592 | 7.158963 | 7.500404 | 7.430118 |
| GRMZM2G114899 | 6.322108 | 6.291861 | 6.044394 | 5.950002 | 6.048977 | 5.847997 | 6.297925 | 6.14037 | 5.830103 | 6.128871 | 5.809672 | 6.331634 | 5.765535 | 6.08151 | 5.790251 | 6.35297 | 6.407523 | 6.014578 | 6.066735 | 6.230549 | 6.200653 | 6.050066 | 5.773205 | 5.71177 | 5.812498 |
| GRMZM2G118939 | 8.566397 | 7.532551 | 7.492975 | 8.225497 | 7.687831 | 8.311748 | 8.436878 | 8.000113 | 8.996106 | 8.699642 | 8.467443 | 8.347267 | 8.470008 | 7.576447 | 8.169324 | 8.767754 | 7.673839 | 7.975561 | 7.623223 | 8.297925 | 8.777781 | 7.572435 | 8.512701 | 8.500882 | 8.315512 |
| GRMZM2G119521 | 6.592008 | 7.983735 | 8.033643 | 8.589651 | 7.32391 | 8.556927 | 6.69265 | 7.48075 | 9.3572 | 6.699052 | 7.86802 | 6.536364 | 8.65814 | 8.11598 | 7.118526 | 6.649759 | 8.047288 | 6.463688 | 8.029895 | 6.972233 | 6.756756 | 6.404971 | 8.19584 | 9.457217 | 8.458407 |
| GRMZM2G121715 | 8.287897 | 6.315784 | 7.65864 | 7.776696 | 6.540709 | 8.3041 | 7.186758 | 7.257765 | 9.134247 | 8.290295 | 7.79967 | 7.778669 | 8.336149 | 8.315512 | 7.155527 | 8.075372 | 6.724514 | 6.721236 | 7.36054 | 7.227568 | 8.163297 | 6.714108 | 8.056909 | 8.509854 | 7.940167 |
| GRMZM2G125308 | 10.90805 | 9.320214 | 7.737078 | 11.09542 | 10.15398 | 11.67324 | 9.767357 | 10.11309 | 11.18651 | 10.43577 | 11.08793 | 10.8159 | 11.65938 | 9.776285 | 10.57239 | 10.38788 | 8.302319 | 10.29459 | 8.140319 | 10.13982 | 10.80242 | 9.298658 | 11.86997 | 11.99259 | 11.39907 |
| GRMZM2G127984 | 4.92505 | 5.78999 | 4.709842 | 5.66249 | 5.111866 | 5.202026 | 5.499846 | 5.099716 | 4.74685 | 4.849499 | 5.102238 | 4.598127 | 5.181103 | 8.783195 | 5.436961 | 4.75809 | 5.332708 | 4.878725 | 4.64501 | 5.850249 | 4.724105 | 4.82324 | 5.431623 | 5.024142 | 5.117695 |
| GRMZM2G132184 | 9.208478 | 8.004052 | 8.755322 | 8.26908 | 8.472691 | 8.803292 | 8.884934 | 8.736233 | 9.713799 | 9.102894 | 8.811471 | 9.246503 | 9.103891 | 8.434211 | 8.636661 | 9.352706 | 8.061614 | 8.109622 | 8.437461 | 9.24103 | 9.140753 | 8.568564 | 8.964918 | 9.052405 | 8.59902 |
| GRMZM2G137468 | 9.027685 | 8.550054 | 8.831687 | 9.044531 | 8.529431 | 9.418464 | 9.039988 | 9.215921 | 9.710462 | 8.819828 | 9.20232 | 8.91582 | 9.42488 | 8.929229 | 9.3376 | 9.130957 | 8.794708 | 9.801531 | 8.818646 | 8.788784 | 9.104232 | 9.353919 | 9.442487 | 9.335033 | 9.366344 |
| GRMZM2G139223 | 3.672425 | 3.791814 | 3.903038 | 4.988685 | 3.802193 | 4.307429 | 3.91934 | 3.845992 | 3.979111 | 3.734439 | 3.604071 | 3.512227 | 4.169123 | 10.01664 | 3.934517 | 3.574102 | 3.792855 | 3.667892 | 3.72465 | 3.707083 | 3.590961 | 3.626439 | 3.956986 | 4.276497 | 3.783457 |
| GRMZM2G140095 | 8.021979 | 7.513807 | 7.359222 | 8.064581 | 7.662704 | 8.025361 | 8.0268 | 8.216697 | 8.126188 | 7.856488 | 8.152589 | 7.905748 | 8.198101 | 7.581502 | 8.476139 | 8.22062 | 7.407013 | 8.792205 | 7.308976 | 8.059561 | 8.168472 | 7.908693 | 8.208137 | 8.331006 | 8.137452 |
| GRMZM2G140590 | 9.244316 | 8.244411 | 8.612242 | 9.689352 | 9.042425 | 9.568545 | 9.201389 | 9.420234 | 10.63089 | 9.890751 | 9.958437 | 9.653168 | 9.919847 | 9.595295 | 9.439062 | 9.469255 | 8.465322 | 8.791554 | 8.576069 | 9.99349 | 9.424187 | 9.637422 | 9.887251 | 10.34554 | 9.532531 |
| GRMZM2G144042 | 8.001014 | 6.353147 | 8.377948 | 7.446174 | 6.63619 | 6.690696 | 7.456888 | 7.679621 | 7.374257 | 7.638726 | 7.773073 | 7.635101 | 6.961044 | 7.137811 | 9.314039 | 8.113586 | 6.67511 | 8.793116 | 7.119875 | 8.076495 | 7.956812 | 8.009269 | 6.840211 | 7.804905 | 6.643856 |
| GRMZM2G147051 | 8.933986 | 7.116448 | 5.35931 | 8.322875 | 8.258802 | 6.215679 | 8.425216 | 8.265193 | 5.434295 | 9.480346 | 8.554934 | 8.003377 | 6.020147 | 5.934988 | 6.971084 | 8.476787 | 5.938991 | 6.131754 | 5.595146 | 6.77215 | 8.18933 | 8.805131 | 6.202026 | 6.05159 | 6.259649 |
| GRMZM2G148962 | 7.365535 | 6.771093 | 6.974759 | 6.989253 | 6.791293 | 7.252382 | 7.598276 | 7.137504 | 7.194658 | 7.410663 | 6.835545 | 7.627753 | 7.270903 | 6.891784 | 6.345538 | 7.596041 | 6.721509 | 6.483655 | 7.095924 | 7.7924 | 7.332618 | 6.70335 | 6.949768 | 6.86839 | 6.999662 |
| GRMZM2G149943 | 8.262659 | 7.153603 | 6.707083 | 7.363171 | 7.347666 | 7.550901 | 7.522621 | 7.723968 | 7.22294 | 8.427355 | 7.458202 | 8.485709 | 7.301953 | 6.910493 | 7.001352 | 8.710049 | 7.306335 | 6.956521 | 6.85997 | 8.187055 | 8.505137 | 7.388103 | 7.385431 | 7.036064 | 7.558191 |
| GRMZM2G150806 | 8.046524 | 8.89721 | 8.302228 | 8.262987 | 8.380504 | 8.131908 | 8.171377 | 8.214076 | 8.084011 | 8.189034 | 8.202124 | 7.858292 | 8.030171 | 8.272816 | 8.27975 | 7.984532 | 9.061965 | 8.082522 | 8.522032 | 8.23998 | 8.01998 | 8.202761 | 8.090377 | 7.975504 | 8.350586 |
| GRMZM2G152901 | 6.889108 | 7.702935 | 7.255312 | 7.569856 | 7.456478 | 7.600879 | 7.134529 | 7.381024 | 7.317413 | 6.904002 | 7.507081 | 6.720552 | 7.476544 | 7.537917 | 7.389137 | 6.61309 | 7.57501 | 7.370426 | 7.392918 | 7.462952 | 6.852623 | 7.098874 | 7.656997 | 7.304055 | 7.813396 |
| GRMZM2G153945 | 5.229588 | 4.852498 | 4.519164 | 5.307793 | 5.665052 | 7.100662 | 5.19101 | 4.959306 | 6.913368 | 5.118941 | 5.279843 | 5.293518 | 6.485588 | 4.613532 | 4.867402 | 5.496335 | 4.831371 | 4.970854 | 4.575917 | 5.302685 | 5.428946 | 4.815575 | 6.674828 | 6.297191 | 7.052785 |
| GRMZM2G157115 | 9.225979 | 8.297971 | 8.952916 | 8.814807 | 8.267817 | 8.198936 | 9.042945 | 8.901138 | 7.756957 | 8.88145 | 9.669913 | 8.79227 | 8.273096 | 8.192441 | 9.044859 | 8.59025 | 8.149188 | 8.818742 | 8.251388 | 8.63205 | 8.841061 | 8.259225 | 8.46785 | 9.595276 | 8.534186 |
| GRMZM2G158045 | 6.676944 | 6.922436 | 7.195446 | 6.875043 | 6.61471 | 6.732269 | 6.6522 | 6.537141 | 6.695715 | 6.714108 | 6.555356 | 6.382321 | 6.697802 | 6.734981 | 6.683837 | 6.480427 | 7.235057 | 6.75208 | 6.843356 | 6.65392 | 6.518378 | 6.593653 | 6.710118 | 6.757557 | 6.647458 |
| GRMZM2G159992 | 10.48734 | 9.935327 | 10.98282 | 10.63334 | 10.03417 | 11.16239 | 10.53586 | 10.59478 | 10.96811 | 10.548 | 10.82633 | 10.58179 | 11.08081 | 10.6371 | 10.09296 | 10.48923 | 10.65976 | 9.970969 | 10.60895 | 10.50062 | 10.34688 | 9.962101 | 11.02477 | 10.93106 | 10.58269 |
| GRMZM2G161380 | 6.923981 | 6.916954 | 6.161283 | 7.099085 | 7.0974 | 6.926237 | 7.080231 | 7.019035 | 6.581351 | 7.182792 | 7.287897 | 7.384913 | 6.755822 | 6.227086 | 7.91259 | 6.963705 | 6.752347 | 8.389997 | 6.062424 | 7.400111 | 7.150357 | 7.300032 | 7.021813 | 6.76195 | 6.99876 |
| GRMZM2G165433 | 4.244126 | 4.313246 | 4.444932 | 5.023255 | 4.302319 | 4.527321 | 4.405312 | 4.246408 | 4.328406 | 4.32337 | 4.095924 | 4.186659 | 4.386121 | 9.517059 | 4.428276 | 4.18428 | 4.403949 | 4.525443 | 4.28762 | 4.217231 | 4.228819 | 4.129283 | 4.263034 | 4.663345 | 4.248687 |
| GRMZM2G166719 | 7.459186 | 7.489687 | 6.629648 | 8.019035 | 6.670019 | 7.862265 | 6.287251 | 6.907251 | 6.649472 | 7.804776 | 7.871043 | 7.068993 | 7.893848 | 6.998985 | 6.543496 | 7.206721 | 7.433961 | 5.794156 | 6.0581 | 7.160577 | 7.100767 | 5.725469 | 7.767258 | 8.107374 | 7.495935 |
| GRMZM2G168416 | 4.898208 | 5.102238 | 4.986411 | 5.045268 | 4.788686 | 5.26341 | 4.87578 | 4.712045 | 5.008989 | 4.887038 | 4.813525 | 4.615887 | 5.023255 | 4.980939 | 5.080231 | 4.843481 | 5.139551 | 4.980025 | 4.901108 | 4.995937 | 4.861955 | 4.82273 | 5.06048 | 5.078524 | 5.250204 |
| GRMZM2G175164 | 5.973382 | 6.511595 | 6.496175 | 6.065874 | 6.024807 | 6.475409 | 6.253044 | 5.867896 | 7.242507 | 5.673839 | 5.791814 | 5.750874 | 6.689299 | 6.551824 | 6.052459 | 5.784242 | 6.761817 | 6.064366 | 6.467769 | 6.555356 | 5.810957 | 5.976593 | 6.281884 | 7.421055 | 6.558268 |
| GRMZM2G177445 | 9.645298 | 6.508429 | 6.281327 | 8.710152 | 7.701272 | 7.751276 | 9.721338 | 8.729485 | 7.327687 | 9.314447 | 8.920085 | 8.891085 | 7.882337 | 6.76288 | 7.873752 | 9.136068 | 6.279471 | 8.298383 | 6.246218 | 8.750975 | 9.073338 | 8.212958 | 8.18844 | 7.763478 | 7.975045 |
| GRMZM2G178787 | 8.907371 | 8.774952 | 7.273982 | 9.44145 | 9.481497 | 9.345006 | 8.372299 | 9.338826 | 9.933219 | 9.310226 | 9.890325 | 9.852904 | 9.466851 | 8.4325 | 8.179959 | 9.528962 | 7.42383 | 6.998308 | 7.454916 | 9.220233 | 9.55386 | 8.917969 | 9.112231 | 9.275473 | 9.175624 |
| GRMZM2G180775 | 6.177719 | 5.564073 | 5.648465 | 5.808385 | 5.806066 | 6.26791 | 5.093391 | 5.596339 | 5.80555 | 6.275752 | 6.979568 | 5.475409 | 6.15765 | 5.607626 | 7.101608 | 5.19456 | 5.312157 | 5.171527 | 5.088735 | 5.530133 | 5.086189 | 6.220717 | 6.264536 | 7.276217 | 6.595444 |
| GRMZM2G181231 | 7.5499 | 7.897785 | 7.934575 | 8.458448 | 7.757823 | 7.998816 | 10.20738 | 7.435629 | 6.568336 | 8.392532 | 8.473016 | 8.566625 | 8.562204 | 7.215873 | 5.479295 | 7.382408 | 7.83788 | 5.456806 | 6.274075 | 10.1962 | 7.230837 | 5.461725 | 8.864248 | 8.65864 | 8.085021 |
| GRMZM2G301647 | 5.740658 | 6.194757 | 5.883132 | 5.731998 | 5.85599 | 5.696272 | 5.644722 | 5.608514 | 5.670444 | 5.650765 | 5.491212 | 5.423914 | 5.699052 | 6.86059 | 5.66021 | 5.555509 | 6.147103 | 5.534809 | 6.019257 | 5.847746 | 5.66306 | 6.011451 | 5.742006 | 5.594847 | 5.68173 |
| GRMZM2G305822 | 7.164203 | 6.291861 | 6.921008 | 7.491292 | 6.886794 | 8.489728 | 7.030998 | 6.961508 | 8.849561 | 7.370251 | 7.742949 | 7.508112 | 8.38806 | 8.162291 | 6.996954 | 6.714658 | 6.380937 | 6.849124 | 6.980482 | 6.965669 | 6.863071 | 6.657783 | 8.157094 | 8.506089 | 8.332037 |
| GRMZM2G317938 | 6.683135 | 5.947432 | 5.800382 | 5.238023 | 5.774787 | 6.177519 | 5.997067 | 5.77426 | 6.661493 | 6.506208 | 6.457627 | 6.808514 | 6.023921 | 6.658069 | 5.849999 | 6.753284 | 5.979339 | 6.386983 | 5.762083 | 5.909533 | 6.669169 | 5.765004 | 5.840211 | 5.704595 | 5.931447 |
| GRMZM2G343024 | 9.67779 | 8.883774 | 9.063584 | 9.632286 | 9.179312 | 9.915521 | 9.511061 | 9.691011 | 9.895878 | 9.656175 | 9.745254 | 9.707929 | 10.01508 | 9.309408 | 9.412041 | 9.494956 | 9.084463 | 9.199452 | 9.096557 | 9.900369 | 9.545621 | 8.7303 | 10.08292 | 9.927319 | 9.801401 |
| GRMZM2G346132 | 5.441616 | 4.60585 | 4.119356 | 6.132782 | 5.170726 | 4.60585 | 4.58376 | 6.18844 | 4.117695 | 5.769507 | 5.656782 | 4.750607 | 4.50716 | 5.992315 | 5.477354 | 4.942515 | 4.546586 | 6.689579 | 4.189825 | 4.474436 | 5 | 6.67101 | 5.011675 | 4.432291 | 4.724105 |
| GRMZM2G355450 | 7.581728 | 6.866166 | 6.65263 | 6.71452 | 6.996841 | 6.752347 | 7.682503 | 7.024142 | 6.961044 | 7.187253 | 6.672567 | 7.640534 | 6.68888 | 6.47476 | 6.773733 | 7.654779 | 6.8166 | 7.101083 | 6.622198 | 6.929318 | 7.551824 | 7.141801 | 6.612942 | 6.809672 | 6.740793 |
| GRMZM2G364172 | 5.720005 | 5.743353 | 5.972003 | 5.911452 | 5.667608 | 6.343763 | 6.06415 | 5.721919 | 6.835671 | 5.674969 | 6.032321 | 5.845992 | 6.360715 | 5.799346 | 6.44791 | 5.90665 | 6.171927 | 6.419033 | 5.869378 | 6.137708 | 5.716442 | 6.856612 | 6.265849 | 6.665194 | 5.888013 |
| GRMZM2G365319 | 4.84147 | 7.184479 | 4.920293 | 8.826294 | 7.227375 | 8.339984 | 5.695715 | 7.418359 | 7.273329 | 7.678354 | 8.372343 | 4.885574 | 8.464096 | 5.112283 | 5.071677 | 4.863938 | 4.869871 | 4.653633 | 4.760753 | 6.386639 | 5.039577 | 4.536675 | 8.058587 | 8.585376 | 8.38297 |
| GRMZM2G378547 | 7.138835 | 5.626439 | 5.920055 | 7.081617 | 5.520108 | 6.528571 | 6.393691 | 7.013462 | 6.091488 | 7.941341 | 7.017365 | 6.526382 | 6.161283 | 11.98435 | 5.806324 | 7.226027 | 5.650765 | 7.528181 | 5.777683 | 6.959422 | 6.659639 | 9.213031 | 5.783195 | 6.499686 | 5.773996 |
| GRMZM2G382104 | 7.765468 | 8.518889 | 8.218345 | 7.920948 | 8.127221 | 8.65159 | 7.911632 | 7.809543 | 7.988798 | 7.707359 | 7.613532 | 7.546431 | 8.255548 | 8.04259 | 8.111657 | 7.544655 | 8.548398 | 7.781884 | 8.460538 | 7.995937 | 7.652558 | 7.975619 | 8.506565 | 8.112231 | 8.917611 |
| GRMZM2G406601 | 7.765468 | 8.518889 | 8.218345 | 7.920948 | 8.127221 | 8.65159 | 7.911632 | 7.809543 | 7.988798 | 7.707359 | 7.613532 | 7.546431 | 8.255548 | 8.04259 | 8.111657 | 7.544655 | 8.548398 | 7.781884 | 8.460538 | 7.995937 | 7.652558 | 7.975619 | 8.506565 | 8.112231 | 8.917611 |
| GRMZM2G433433 | 6.854744 | 7.140267 | 6.982765 | 7.642774 | 6.781884 | 7.098137 | 6.87209 | 7.245363 | 6.891905 | 6.998647 | 7.026025 | 6.859348 | 7.082788 | 6.782671 | 5.879216 | 6.805034 | 6.945444 | 5.308521 | 6.945912 | 7.032321 | 6.80271 | 5.952334 | 7.106746 | 6.399342 | 7.004389 |
| GRMZM2G471779 | 8.887342 | 7.135042 | 8.200408 | 8.672107 | 8.08672 | 7.876333 | 8.607404 | 8.402074 | 8.510052 | 9.138477 | 8.667679 | 8.878296 | 8.063988 | 7.763412 | 8.449644 | 9.068429 | 7.101188 | 8.398444 | 8.12003 | 8.599429 | 8.937903 | 8.80526 | 8.101608 | 8.410621 | 7.863195 |
